# Preparation of a phosphotyrosine-protein standard for use in semiquantitative western blotting with enhanced chemiluminescence

**DOI:** 10.1371/journal.pone.0234645

**Published:** 2020-06-18

**Authors:** Nancy Kendrick, Ginny Powers, Jon Johansen, Matt Hoelter, Andrew Koll, Sofia Carlson, Devika Channaveerappa, Costel C. Darie

**Affiliations:** 1 Kendrick Labs, Inc, Madison, WI, United States of America; 2 Biochemistry & Proteomics Group, Department of Chemistry & Biomolecular Science, Clarkson University, Potsdam, NY, United States of America; Duke University School of Medicine, UNITED STATES

## Abstract

Protein tyrosine phosphorylation is key to activation of receptor tyrosine kinases (RTK) that drive development of some cancers. One challenge of RTK-targeted therapy is identification of those tumors that express non-mutated but activated RTKs. Phosphotyrosine (pTyr) RTK levels should be more predictive of the latter than expressed total protein. Western blotting (WB) with a pTyr antibody and enhanced chemiluminescence (ECL) detection is sufficiently sensitive to detect pTyr-RTKs in human tumor homogenates. Presentation of results by comparing WB images, however, is wanting. Here we describe the preparation of a new pTyr-protein standard, pTyr-ALK48-SB (pA), derived from a commercial anaplastic lymphoma kinase (ALK) recombinant fragment, and its use to quantify pTyr-epidermal growth factor receptor (pTyr-EGFR) in commercial A431 cell lysates. Linearity of one-dimensional (1D) WB plots of pA band density versus load as well as its lower level of detection (0.1 ng, 2 fmole) were determined for standardized conditions. Adding pA to two lots of A431 cell lysates with high and low pTyr-EGFR allowed normalization and quantification of the latter by expressing results as density ratios for both 1D and 2D WB. This approach is semi-quantitative because unknown RTKs may be outside the linear range of detection. Semiquantitative ratios are an improvement over comparisons of images without a reference standard and facilitate comparisons between samples.

## Introduction

Receptor tyrosine kinases (RTK) such as epidermal growth factor receptor (EGFR) are large, transmembrane proteins that function in signal transduction. Binding of a serum ligand (EGF for example) to an extracellular protein domain triggers protein dimerization and subsequent trans-phosphorylation of multiple tyrosine residues on intracellular kinase domains. The RTK phosphotyrosines (pTyr) plus adjacent amino acids become docking sites for matching Src homology 2 domains on cytosolic proteins. The latter in turn interact to cause cell growth and differentiation. Tyrosine phosphorylation is the key event leading to RTK activity, not protein expression per se. Aberrant pTyr-RTK activity sometimes drives cancer growth [1, 2].

**Fig 1:**
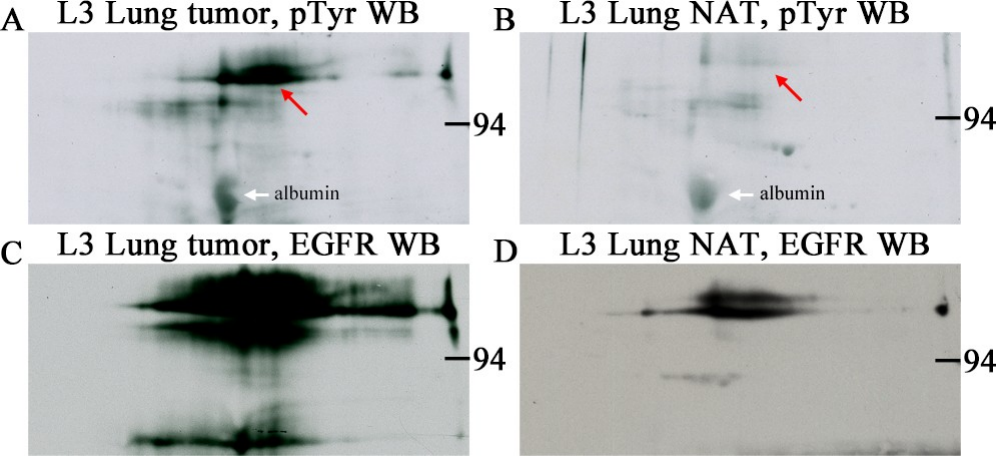
Phosphotyrosine 2D WB signal detected in human lung tumor and normal adjacent tissue samples comigrates with EGFR signal. Phosphotyrosine (A and B) and EGFR (C and D) 2D western blots from human lung tumor (squamous cell carcinoma, A and C) and normal adjacent tissue (NAT, B and D) samples purchased from a tissue bank, Bio-IVT. Sample load was 200 μg total protein for each of the four 2D gels. The pTyr-protein signal (red arrows) was strong in the tumor tissue and faint in NAT and co-migrated with the EGFR signal in both. The heavily glycosylated EGFR protein gives a large blurry spot profile due to glycan microheterogeneity. Samples were identically prepared by homogenization in SDS buffer with heating. White arrows indicate nonspecific binding to albumin, an abundant protein that serves as a 2D pI/MW marker.

Preliminary results in our laboratory suggested that standardized 1- and 2-dimensional sodium dodecyl sulfate polyacrylamide gel electrophoresis (1D and 2D SDS PAGE, 1D and 2D) in combination with pTyr western blot (WB) with enhanced chemiluminescence (ECL) detection is sensitive enough to directly detect pTyr-RTKs in excised human lung tumors. While 1D pTyr WB was useful for screening to find tumor samples of interest, the broad 1D bands were of little use for identification. Comparing 2D WB patterns, where one sample is loaded per gel, worked better for identifying the high molecular weight pTyr-RTKs as shown in Fig 1. In this figure the 2D pTyr WB pattern of a human squamous cell carcinoma lung tumor sample and its control are compared to that of pTyr -EGFR WBs from the same samples. Increased glycosylation with elevated sialyation is a characteristic of the metastatic cell phenotype [3, 4]; glycan microheterogeneity is likely causing the 2D spots to be large and diffuse.

That brought an exciting possibility to mind: pTyr WB might predict patient response to RTK inhibitor drugs. Patients whose tumor showed a strong pTyr-RTK signal, pTyr-EGFR for example, would likely respond to the corresponding inhibitor, e.g. Necitumumab, a WT EGFR inhibitor [5]. This approach might be directly useful for precision medicine testing, or more likely indirectly useful for finding RNA-seq fingerprints for a predictive genomic assay. For example, Finotello and Trajanoski recently reviewed computational methods for deconvoluting bulk tumor RNA-seq data to predict the composition of tumor-infiltrating immune cells based on immune-specific marker genes or expression signatures [6]. Perhaps a deconvolution approach could be applied to semi-quantify pTyr-RTK driver pathways in lung cancer samples based on pTyr-protein markers. Chang et. al found that RNA fingerprints from a set of 374 house-keeping genes (HKG) could be used to distinguish 6 normal lung tissue samples from 18 lung cancers including three subtypes [7]. HKG expression fingerprints gleaned from tumors known to have pTyr-RTK proteins might be an alternative method for predicting the presence of the latter.

One major problem with WB, however, is that pictorial results from even small group of samples are cumbersome to tabulate and present. This is especially true for 2D WBs where only one sample is applied per blot. Light emission by the ECL reagent varies with time after application, and also with exposure time [8] making quantification difficult. The linear relationship between signal intensity and sample must be confirmed for WBs loaded with equal protein [9]. An internal standard that could be used to normalize pTyr WB results for comparisons between different days was needed to go forward.

Our goal was to quantify results from the PY20 monoclonal pTyr Ab [10] western blots of tumor whole cell lysates such as those in Fig 1. Total protein load for whole cell lysates is kept constant, 40 and 200 μg for 1D and 2D gels respectively as recommended by Fosang and Colbran [9]. The reactive pTyr proteins are in very low amounts that vary greatly between the samples. They are not visible with any staining method and are only detectable by WB. Normalizing the pTyr signal to individual internal control proteins such as α-tubulin or ß-actin or to total protein loaded is inappropriate since the pTyr signal varies with exposure time to the same blot. Expressing results as density ratios of unknown pTyr-proteins to that of a known pTyr-protein standard present in the same gel seemed the best way to quantify results. The unknowns would be varying in tandem with the standard pTyr-protein signal for the same set of conditions. In that case pTyr WB results could be presented as numbers (density ratios) rather than pictures. To our knowledge, no suitable commercial pTyr-protein standards are available.

Thus, we have developed and characterized a pTyr-protein standard, pTyr-ALK48 (pA), of molecular weight 48 kDa that contains at least two phosphotyrosine residues as evidenced by mass spectrometry (MS). In this paper we present a feasibility study of the usefulness of pA that includes linearity of 1D and 2D WB response, normalization of different film exposures, and comparisons of density ratios of two lots of commercial A431 cell lysates containing different amounts of pTyr-EGFR. This approach is semi-quantitative because unknown RTKs may be outside the linear range of detection, the number of pTyr residues per unknown protein may vary, and the amount of inert protein, serum or media contamination for example, may vary. Semiquantitative measurements are a great improvement, however, over pictorial comparisons without any reference standard.

## Results

Preliminary tests of two commercially available pTyr-proteins, c-Jun N-terminal kinase 1 (JNK1) and chemically tyrosinylated bovine serum albumin (pTyr BSA) were discouraging (S1 and S2 Figs). The JNK1 protein resolved well into three isoforms detectable by Coomassie staining, but only one minor isoform showed tyrosine phosphorylation. This standard would have been too costly. The pTyr-BSA gave a diffuse 1D signal that was not quantifiable. For the sake of reproducibility and quantification, an ideal standard should have high tyrosine phosphorylation levels and minimal streaking.

Development of a new pTyr-standard by in-house phosphorylation of a recombinant protein seemed the only way forward. The standard should run as a discrete quantifiable 1D band or 2D spot and be less than ~60 kDa so that it is clearly distinct from high MW RTKs on WB patterns. It should be concentrated enough to add to every 2D sample, or run on every 1D blot, for the purpose of normalization of results.

### A recombinant fragment of a known RTK was the starting material

Anaplastic lymphoma kinase (ALK) is a much-studied RTK thought to play a role in development of the nervous system. A commercially available *Escherichia coli* recombinant ALK fragment containing the kinase domain has a mass of 47,991 Da (S3 Fig; blue sequence). This product, ALK48, was used as the starting material for a pTyr protein standard.

### The tyrosine kinase reaction was successfully carried out in vitro

ALK48 is offered by ProQinase (Freiburg, Germany) as a tool for testing the efficacy of ALK inhibitors. To achieve auto-Tyr phosphorylation, this fragment was incubated in an ATP-containing reaction mixture specified by ProQinase for 60 min at 30°C. Protein aliquots collected before and after the kinase reaction were analyzed by 2D pTyr WB to see if the protein had acquired pTyr residues.

**Fig 2:**
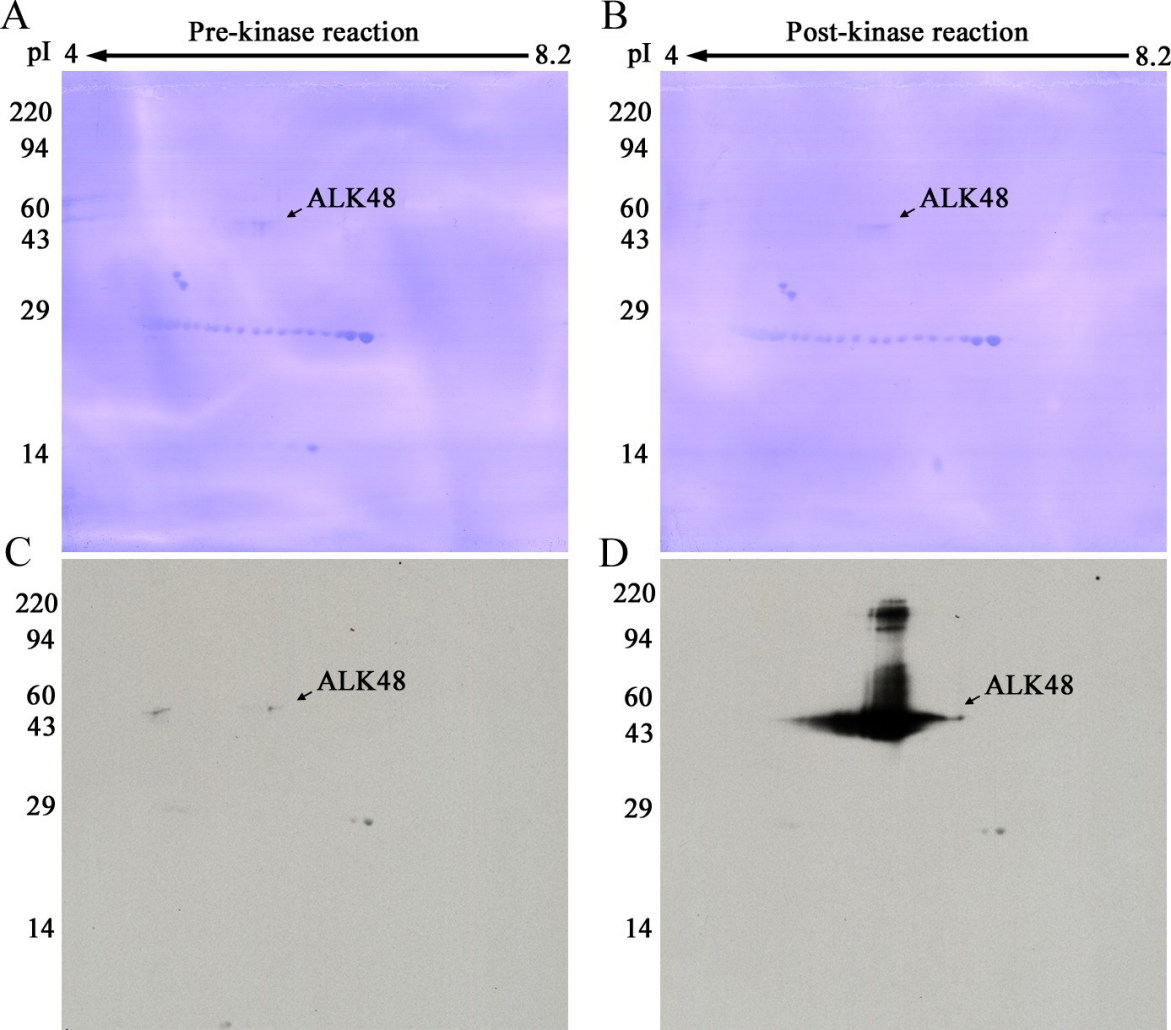
**2D PVDFs (A, B) and pTyr WBs (C, D).** ALK48 protein pre- (A, C) and post- (B, D) kinase reaction. A and C are from a 2D gel loaded with 1 μg ALK48. B and D are from an identically run second gel loaded with 1 μg of the same protein after the kinase reaction. Western blots are both from 3-min Kodak MR film exposures. Carbamylated carbonic anhydrase pI markers were added. Molecular weight markers are shown on the left.

Fig 2 shows the pTyr western blotting pattern obtained with the PY20 antibody for 2D gels loaded with 1 μg ALK48 before (Fig 2A) and after (Fig 2B) the kinase reaction. The tyrosine autophosphorylation clearly was successful. The PY20 antibody binding was essentially zero before and pronounced after the in vitro reaction.

### Alkylation of pTyr-ALK48 cysteines reduced 2D gel streaking

**Fig 3:**
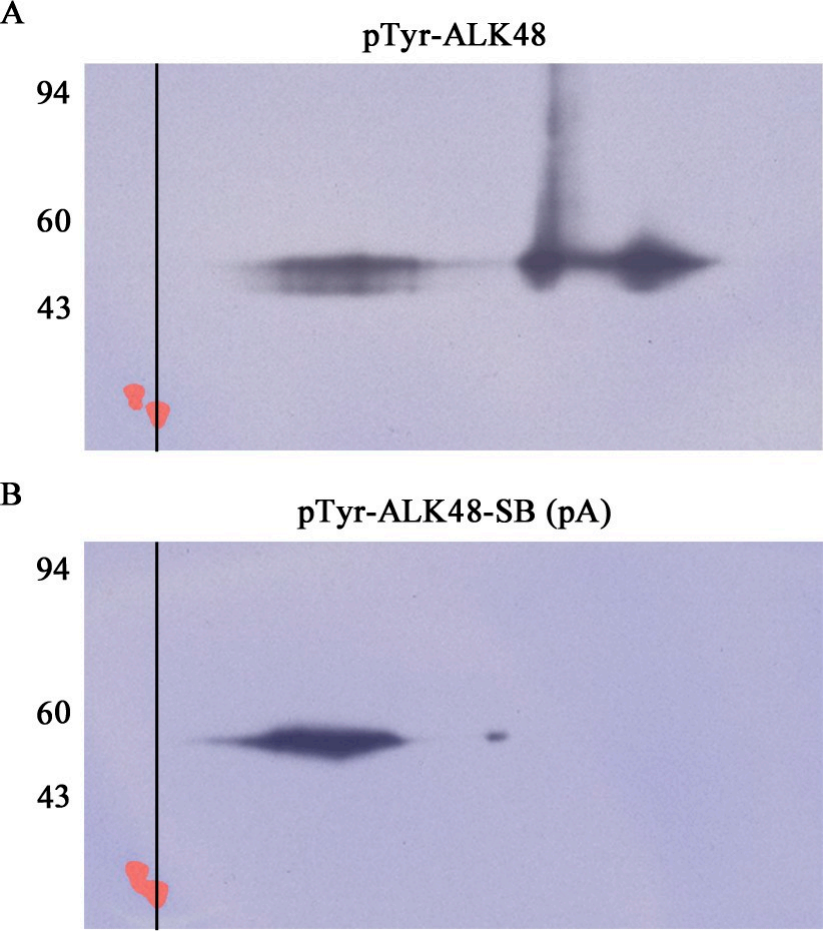
**2D SDS PAGE WBs of pTyr-ALK48 before (A) and after (B) sulfhydryl blocking by alkylation with IAA.** Each WB film image has been superimposed over its corresponding Coomassie blue stained PVDF blot image so that the tropomyosin standard spots, pI 5.2, MW 33 kDa, visible only by Coomassie staining, can be aligned via the vertical line. The Coomassie tropomyosin spots were colored red in Adobe Elements. The alkylation reaction caused the multiple charge forms observed in 2A to condense into a major and minor 2D spot with reduced streaking in 2B. Each 2D gel was loaded with 20 ng of pTyr-ALK48.

Spot spreading shown in Fig 2B for the heavy load of 1 μg is normal. Even at a much lower load of 20 ng, however, the pTyr-ALK48 protein streaked in a non-reproducible fashion as exemplified in Fig 3A. Irreproducible horizontal streaking would make this protein a poor 2D internal standard.

During the overnight IEF step, SDS in the sample gets stripped off proteins by a nonionic detergent, IGEPAL, present in the tube gel [11]. One reason for 2D streaking of pTyr-ALK48 might be transient protein aggregation as polypeptide chains lose SDS. Kinase domains of dimerized RTKs have enough affinity for each other in vivo to allow trans-tyrosine phosphorylation to occur. Disufide bonding between cysteines is known to be a factor in protein aggregation [12]. The pTyr-ALK48 fragment contains 10 cysteines. If disulfide bonding is involved, then blocking the cysteines might reduce streaking. To test this hypothesis, an alkylation reaction with iodoacetamide (IAA) was used to block cysteine sulfhydryl groups. Fig 3 shows 2D pTyr WBs run before and after the IAA reaction. The post-reaction 2D image in Fig 3B shows the goal was accomplished. The alkylation condensed the pTyr-ALK48 protein into a single 2D spot, albeit somewhat streaky. The final iodoacetylated (sulfhydryl blocked, SB) pTyr standard, pTyr-ALK48-SB, is abbreviated from here on as pA.

Although the pA spot in Fig 3B is still horizontally streaky, it’s much tighter than the non-sulfhydral blocked pTyr-ALK48 and is quantifiable. The position of pA at 48 kDa is distant from that of pTyr-RTKs on 1D and 2D gels which typically run at 100–200 kDa, so any possible interference can be excluded. Therefore, pA is a good pTyr protein candidate to be used as an internal standard for quantification of phosphorylation of pTyr-RTKs as well as most other pTyr-proteins.

### Tyr phosphorylation was confirmed by MS

**Fig 4:**
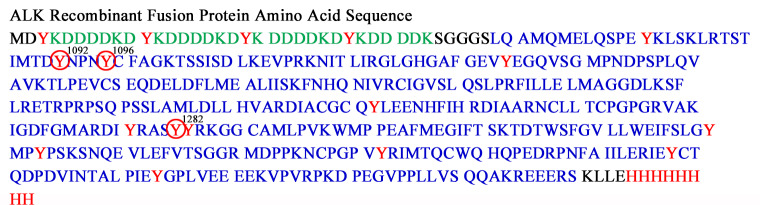
Tyrosine residues Y1096 and Y1282, and to a lesser extent Y1092 are phosphorylated on the pA fragment during the kinase reaction. A red circle indicates a phosphorylated tyrosine residue. Green indicates the Flag tag residues. Red indicates HIS6 tag and tyrosine residues (Y). Blue indicates the ALK fragment residues. Three μg of protein was run on the 1D gels used for pA band cutting.

To determine which tyrosine residues were phosphorylated during the kinase reaction, pA protein bands were cut from a 1D SDS PAGE gel for analysis by MS. Two Tyr, Y1096 and Y1282, were phosphorylated (Fig 4) as demonstrated by identification of the following peptides in which C(#) is a cysteine modified by acrylamide (propionamide), pY is a phosphorylated tyrosine residue, m is oxidized methionine and m/z is the mass to charge ratio: TSTIMTDYNPNpYC(#)FAGK (m/z 1039.03(2+)) and TSTImTDYNPNpYC(#)FAGK (m/z 1047.03(2+)) which had Y1096 phosphorylated and ASYYRK (m/z 434.26(2+)), which had Y1282 phosphorylated. In addition, we also found evidence that Y1092 is phosphorylated, but at a lesser extent than Y1096. Specifically, we found a peptide that corresponds to peptide TSTIMTDpYNPNYC(#)FAGK (m/z 1039.01(2+)). The MS and MS/MS spectra of the three phosphorylated peptides are shown in S4, S5 and S6 Figs.

Indication that some Tyr residues were not phosphorylated was also observed. Specifically, we found a peptide with Y1096 phosphorylated, but the same peptide also contained Y1092 unphosphorylated. In addition, we found a peptide that contained Y1282 phosphorylated, but the same peptide also contained Y1283 unphosphorylated. Furthermore, we found peptide GLGHGAFGEVYEGQVSGmPNDPSPLQVAVK (m/z 1019.60 (3+)) that contained Y1031 unphosphorylated and peptides MDPPKNCPGPVYR (m/z 515.65(3+)), mDPPKNCPGPVYR (520.98(3+)), NCPGPVYR (m/z 488.81(2+), which contained Y1350 unphosphorylated.

### Evidence for usefulness of pA as a pTyr WB standard on 1D gels

Western blotting is a multistep procedure with several critical reagents including primary and secondary antibodies. Multiple pTyr-proteins may be present simultaneously in biological samples in varying amounts. Since both assay and samples have potential for variability, adding a positive control would be useful for method validation as well as normalization of results.

#### WB response is linear for pA band density versus load

The utility of pA as a positive control was tested using 1D SDS PAGE gels loaded with a pA standard curve. In addition, a commercially available pTyr-EGFR standard protein (pE) was purchased and used as a second control to verify spot density ratios. The latter consisted of lysates from A431 cells treated with EGF to generate high levels of pTyr-EGFR.

**Fig 5:**
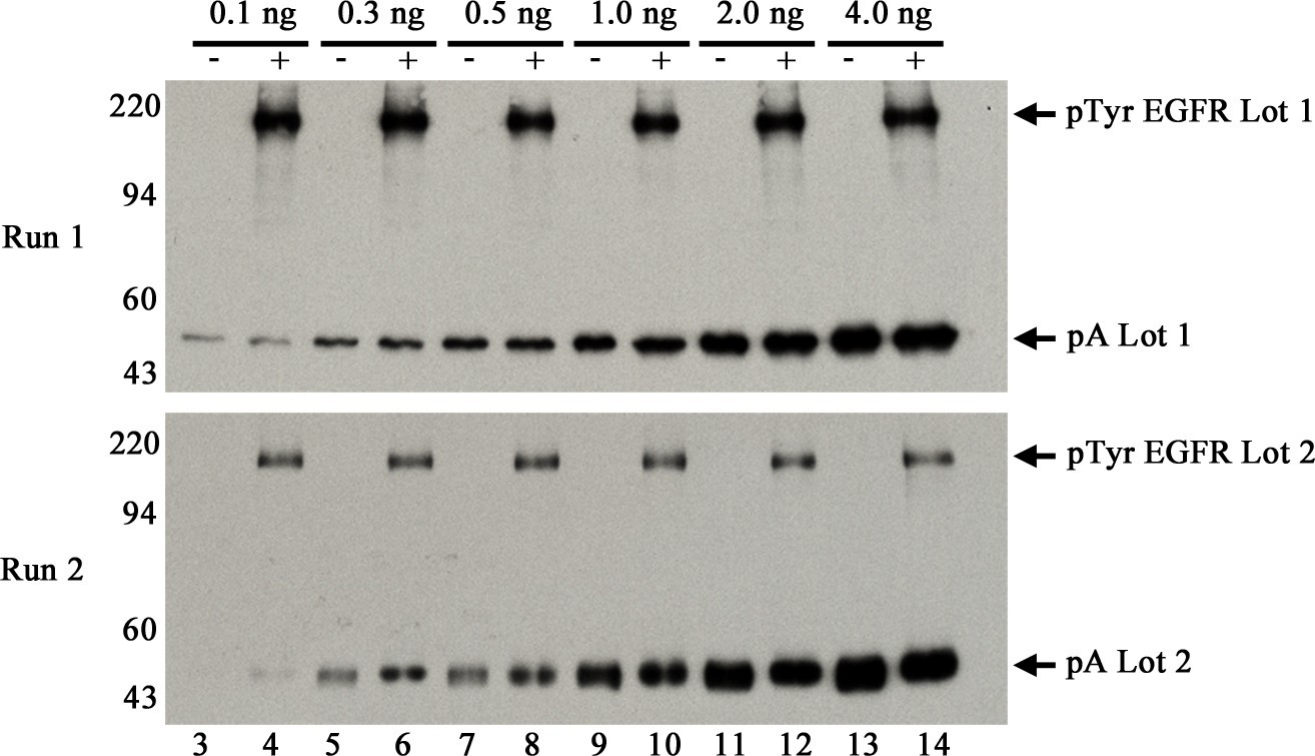
Representative images of 1D pTyr WBs run on separate days in triplicate (Runs 1 and 2). Both sets were loaded with increasing amounts of pA (Lot 1 for Run 1 and Lot 2 for Run 2) as follows: lane 3–4, 0.1 ng; lanes 5–6, 0.3 ng; 7–8, 0.5 ng; 9–10, 1.0 ng; 11–12, 2.0 ng, and 13–14, 4.0 ng. Run 1 gels were loaded with 5 μl pE Lot 1 in even lanes; Run 2 gels were loaded with 5 μl pE Lot 2 in even lanes. Proteins in the 1D gels were transblotted onto PVDF followed by pTyr WB as described in methods. Three and 10-min films were obtained for each WB; 10-min films from the second gel for each run are shown.

Specifically, for two lots of pA, 0.1, 0.3, 0.5, 1.0, 2.0 and 4.0 ng were loaded in duplicate lanes across triplicate 1D gels for two different runs. For pE, two lots containing different concentrations of pTyr-protein were used, Lot 1 and Lot 2. Total protein for pE was low, unknown for Lot 1 and 1 μg/μl for Lot 2 as verified by faint Coomassie stained PVDF patterns. Five μl of pE Lot 1 was loaded in even lanes for Run 1; 5 μl of pE Lot 2 was similarly loaded for Run 2. Fig 5 shows typical pTyr WB images from each run. The pTyr-EGFR bands at ~180 kDa are considerably darker in Lot 1 than Lot 2. The pA patterns from the two runs are similar. The lower limit of WB detection for pA is 0.1 ng or 2 femtomoles. To our knowledge, this is the best sensitivity achieved so far for detection of any pTyr-protein by any method.

**Fig 6:**
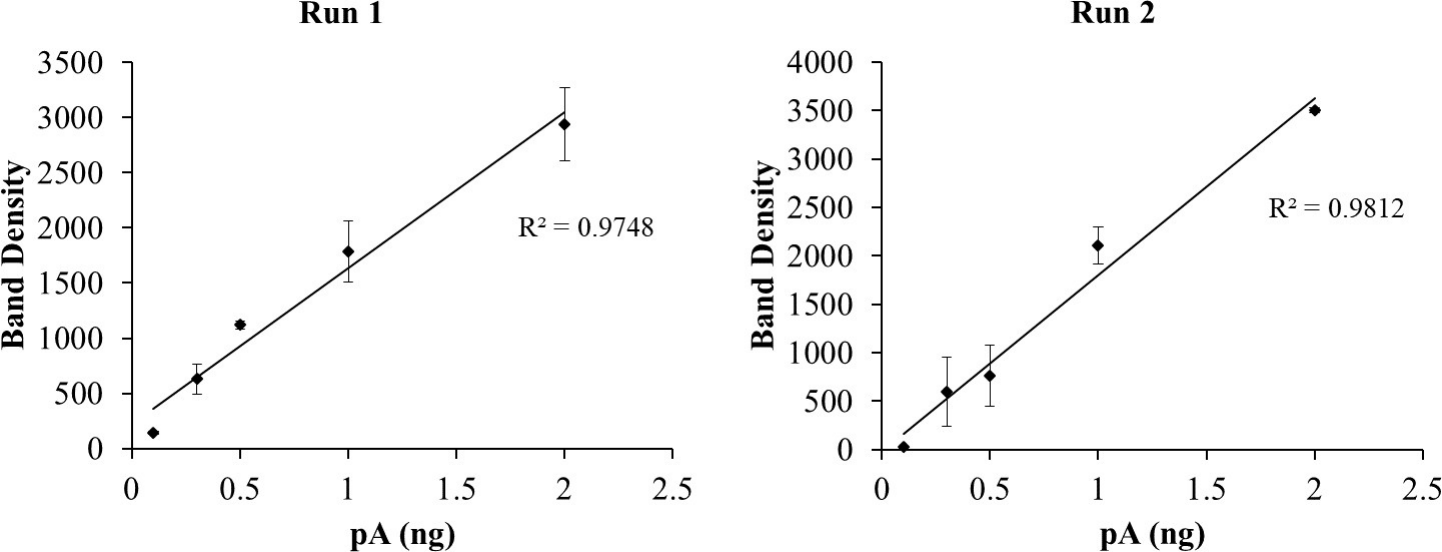
Plots of pTyr band density versus ng pA loaded for WBs shown in Fig 5. The 4-ng point, in the saturated region of the curve, was omitted. All points are n = 2 lanes except the 0.1 ng point in Run 2 for which n = 1.

The 10 min WB films above were scanned with a calibrated densitometer verified to be linear over 0–3 Optical Density units. Band densities on each image were determined using TotalLab software. The pA values from duplicate lanes for each point were averaged before plotting results shown in Fig 6. The 4-ng points (lanes 13 and 14 in Fig 5), in the saturated part of the curve, were omitted. Note that while film has a narrower linear range than CCD cameras, white light densitometers used to quantify films are much easier to validate. Processing multiple WBs in sets using film and scanning later is more efficient than scanning each immediately with a camera.

Fig 6 shows typical plots of pA band density versus load (duplicate lanes averaged) for the two 1D WBs shown in Fig 5. The Excel linear regression trend line has an R^2^ value of 0.9748 for Run 1 and 0.9812 for Run 2. Average R^2^ values ± SD for 3- and 10-min films in triplicate are: Run 1, 3-min: 0.9939 ± 0.002, 10-min: 0.9663 ± 0.008; Run 2, 3-min: 0.9860 ± 0.007, 10 min: 0.9893 ± 0.007. R^2^ average for all (n = 12) was 0.9839 ± 0.012. The closeness of these R^2^ values to 1, a perfect correlation, indicates a nearly linear WB response over the 0–2 ng pA range for the conditions described in Methods.

#### Density ratios normalize film exposures and facilitate comparison of samples

Images of pTyr WBs provide an overview of the number and degree of tyrosine-phosphorylated proteins in a complex sample. However, presenting results from multiple samples run on different days as pictures with arrows is confusing. Expression of pTyr-protein signals as pX/pA band ratios would theoretically be independent of pictures, a huge advantage.

To test this approach the density of pE bands (n = 6 lanes) for 3 and 10 min exposures of all WBs were measured and density ratios calculated relative to the 1 ng pA load (n = 2 lanes). Detailed band density and ratio results from the two representative WBs shown in Fig 5 are provided in S1 Table.

Table 1 below summarizes results for the triplicate WBs (2 films each) per run. The dramatic difference in signal between long and short exposure times is normalized by expressing results as density ratios. On average, Lot 1, with a pE/pA ratio of 1.56, has 6.5 times more pE than Lot 2, ratio 0.24.

**Table 1:** Average pE/pA band density ratios for triplicate WBs from Run 1, (pE and pA Lot 1) and 2 (pE and pA Lot 2) loaded as shown in Fig 5.

1D WB	Run 1: Average pE/pA Ratio		Run 2: Average pE/pA Ratio	
	3 min	10 min	3 min	10 min
gel 1	1.62 ±0.26	1.72 ±0.25	0.21 ±0.02	0.3 ±0.01
gel 2	0.85 ±0.36	1.17 ±0.28	0.13 ±0.02	0.26 ±0.02
gel 3	2.03 ±0.44	1.98 ±0.25	0.20 ±0.01	0.32 ±0.02
Average	1.50 ±0.61	1.63 ±0.43	0.18 ±0.04	0.29 ±0.03
Average All	1.56 ±0.52		0.24 ±0.07	
CV	33		29	

Ratios were obtained by dividing pE WB band densities from lanes 4, 6, 8, 10, 12 and 14 by the 1 ng pA average band density (lanes 9 and 10) on the same gel. Relative to the internal pTyr standard pA, Lot 1 of the commercial pE standard contains 6.5 times more pTyr-EGFR than Lot 2. Both lots were A431 cultured cell lysates that had been treated with EGF to induce the formation of pTyr-EGFR. Coefficient of variation (CV) = SD/mean*100.

### Evidence for usefulness of pA as a pTyr WB standard on 2D gels

The method of 2D SDS PAGE (2D) separates proteins first by charge, according to isoelectric point, then by molecular weight. The final pattern, a starburst rather than a ladder, gives added resolution to crowded 1D areas where a band is comprised of several proteins crowded together. For the 2D method variation used here, the first dimension, isoelectric focusing, is accomplished in acrylamide tube gels which are compatible with SDS buffer [13, 14]. The same samples dissolved in SDS buffer for analysis on 1D gels, can be further resolved on 2D gels which is especially useful for membrane-bound RTKs such as EGFR.

Only one sample is loaded per 2D gel, so experimental design to test the usefulness of pX/pA ratios is necessarily different. The following experiment was devised. Two loads of pA, 1 ng and 2 ng, both in the linear range for standardized 1D WB were loaded along with two fixed amounts, 10 and 20 μl, of pE Lots 1 and 2 on 2D gels according to Table 2.

**Table 2:** Loading conditions for Lots 1 and 2 of positive control pE and the pA standard.

Ratio = X	Ratio = 2X	Ratio = 0.5X	Ratio = Xd
pE/pA	2pE/pA	pE/2pA	2pE/2pA
10 μl pE	20 μl pE	10 μl pE	20 μl pE
1 ng pA	1 ng pA	2 ng pA	2 ng pA

The 1 and 2 ng load of pA are in the linear range of both 3- and 10-min film exposures for 1D plots (Fig 6). Xd = X doubled where pE and pA loads are both doubled to provide a different measurement for X.

**Fig 7:**
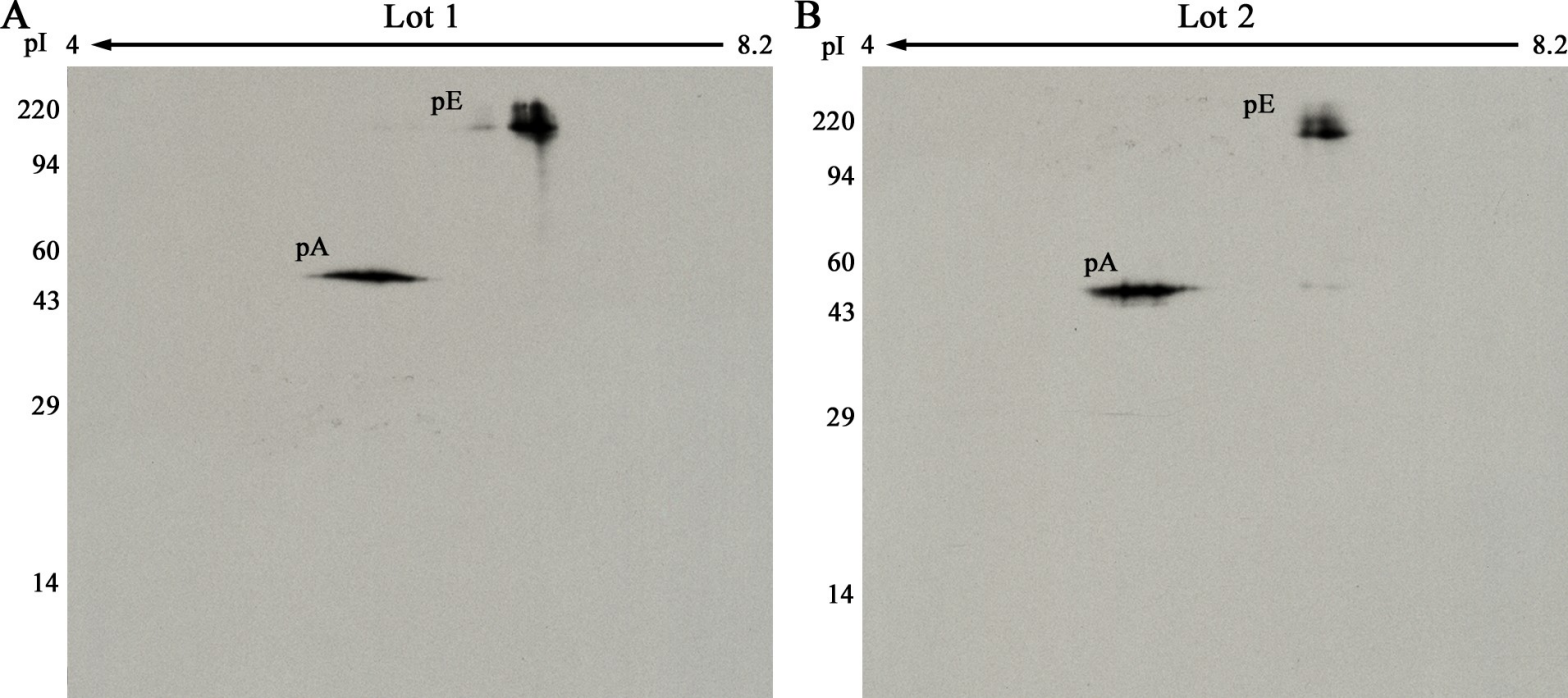
**2D pTyr WB with 20 μl of pE Lot 1 (A) or Lot 2 (B) plus 2 ng of pA.** The molecular weight range is shown on the left, direction of isoelectric focusing on the top. The pE spot is fuzzy because of charge and molecular weight heterogeniety due to glycosylation [15].

Duplicate 2D gels were run and subjected to pTyr WB for each of the four ratios: X, 2X, 0.5X and X double (Xd) for each lot of pE and pA. That is, one set of eight 2D gels was run with pE and pA Lot 1, while a second set of eight 2D gels was run with Lot 2 (Fig 7). If the normalization is successful, a plot of measured pE/pA spot density ratios versus the known loaded pE/pA ratio will be a straight line.

Results from all the 2D gel images in both sets are presented in Table 3. To obtain these results, the 3- and 10-min films were scanned with a calibrated laser densitometer, and images aligned using SameSpots software from TotalLabs. Spot outlines were propagated to all aligned images in the set. Integrated density values above background within the outlines were obtained using SameSpots software. The background subtraction algorithm used was average-on-boundary.

**Table 3:** pE/pA ratio results in duplicate from quantitative analysis of pE Lots 1 and 2 using 2D pTyr WB.

Loaded Ratio	Run 1, Lot 1			Run 2, Lot 2		
	3 min pE/pA (N = 2)	10 min pE/pA (N = 2)	Average pE/pA (N = 4)	3 min pE/pA (N = 2)	10 min pE/pA (N = 2)	Average pE/pA (N = 4)
X	1.86 ±0.66	1.47 ±0.72	1.67 ±0.61	0.54 ±0.03	0.48 ±0.10	0.51 ±0.07
2X	4.03 ±1.92	3.28 ±1.26	3.66 ±1.40	0.80 ±0.24	0.95 ±0.27	0.88 ±0.22
0.5X	0.72 ±0.28	0.71 ±0.22	0.71 ±0.21	0.21 ±0.15	0.19 ±0.07	0.20 ±0.10
Xd	2.06 ±0.47	1.82 ±0.26	1.94 ±0.34	0.47 ±0.04	0.65 ±0.07	0.56 ±0.11

Each loading scheme (Table 2) was run once with pE and pA Lot 1 and once with Lot 2. Each mean includes ±SD.

Results shown in Table 3 indicate that expression of 2D pTyr-WB results as a ratio serves to normalize differences in film exposure times, as it did for 1D. Although the absolute spot densities vary more than two-fold between exposures (not shown), the ratios are similar.

**Fig 8:**
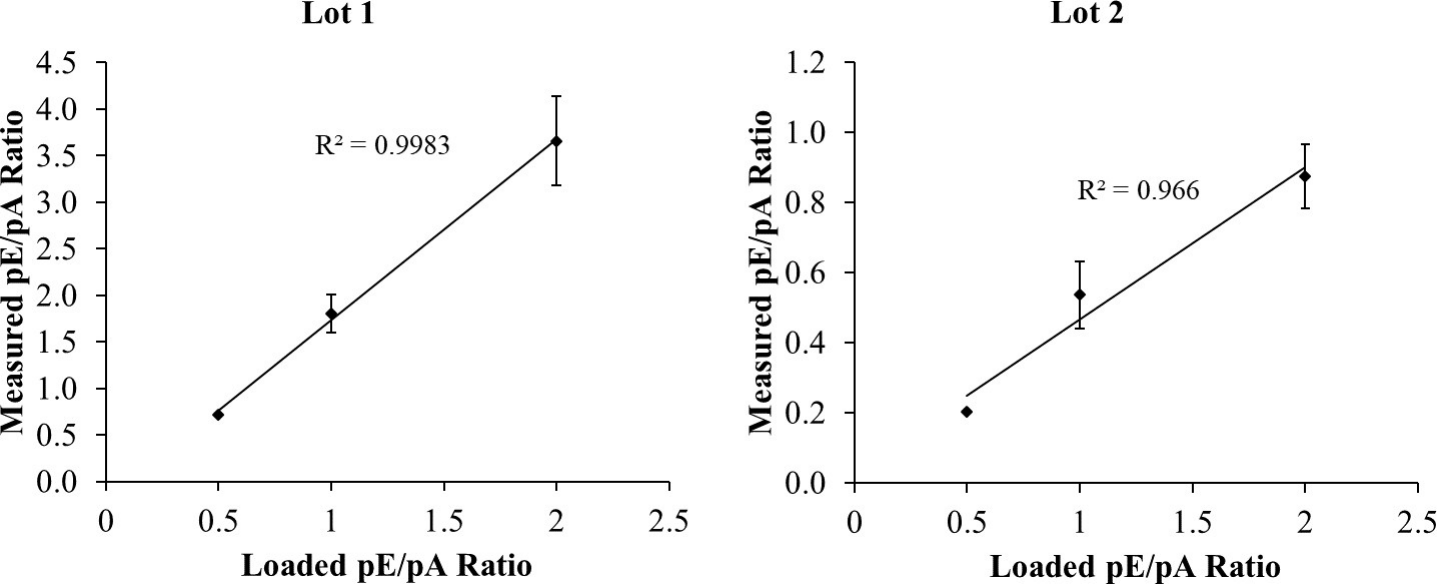
Plots of average measured pE/pA density ratios vs. loaded density ratio. Measured ratios are taken from 3-min and 10-min WBs of eight 2D gels each for pE lots 1 and 2. N = 4 for the 0.5 and 2.0 points, and 8 for the 1.0 point (X and Xd). Error bars show ± 1 SD.

Fig 8 shows plots of loaded (Table 2) versus measured (Table 3) pE/pA density ratios. The plots are linear with R^2^ values >0.966. The linearity of the results indicates that expression of pTyr WB results as a ratio to the internal standard pA normalizes differences in film exposure times for 2D gels, as it did for 1D.

Finally, Table 4 shows quantitative results for the fold difference of pTyr-EGFR in Lots 1 and 2 of the pE standard. Although the SD and CV are relatively high, about 30%, the linearity of the plots for both lots of pE indicates the method is useful for at least semi-quantification of samples.

**Table 4:** Normalized X Ratio results from quantitative 2D analysis of Lots 1 and 2 of pE.

Loaded Ratio	pE Lot 1	pE Lot 2
	X Value (N = 4)	X Value (N = 4)
X	1.67 ±0.61	0.51 ±0.07
2X	1.83 ±0.70	0.44 ±0.11
0.5X	1.43 ±0.41	0.40 ±0.19
Xd	1.94 ±0.34	0.56 ±0.11
Average (N = 16)	1.71 ±0.52	0.48 (±0.13)
CV	30	28

To facilitate comparability, the 2X pE/pA values shown in Table 3 were halved, while the 0.5X values were doubled. Average X values ±SD indicate that Lot 1 contains 3.6 times more pE than Lot 2.

### Ratios of pE/pA allow comparison of pE levels in Lots 1 and 2

The 1D SDS PAGE pTyr WB results shown in Table 1 show Lot 1 contained on average 6.5 times more pE than Lot 2. The 2D SDS PAGE pTyr WB ratios in Table 4 showed Lot 1 contained 3.6 times more pE than Lot 2. These values are within 1 SD of the measurements for the two methods.

## Discussion

A post translational modification, tyrosine phosphorylation at multiple sites, causes activation of all RTKs. In preliminary experiments we found that pTyr WB was sensitive enough to detect putative pTyr-EGFR protein in human tumors (Fig 1). Presentation of pictorial results from multiple analyses, however, was difficult. Here we show that adding a known pTyr-protein standard (pA) to every sample allows unknown pTyr-proteins to be reported as density ratios of 1D band or 2D spots, i.e. pX/pA. Such density ratios are independent of exposure times and facilitate sample comparisons as shown in Table 4 for two lots of cultured A431 cell lysates containing different amounts of pTyr-EGFR.

### pTyr WB is quite sensitive

The reference amount of pA used here, 1 ng (21 femtomoles), was in the middle of the linear region of 1D standard curves and gave a clearly discernable spot on 2D gels. This amount rivals the sensitivity of MS in detection of synthetic pTyr-peptides spiked into adenocarcinoma cultured cell lysates after protein digestion [16, 17]. The lower limit of pA detection by 1D WB was 0.1 ng or 2 femtomoles. To our knowledge, this is the lowest pTyr-protein detection limit reported to date. For practical purposes, the lower limit of detection depends also on the percent recovery of pTyr-RTK from a cell lysate. Membrane protein recoveries are likely high in tissue samples homogenized in SDS buffer with heating until the solution clarifies.

#### CCD camera versus X-ray film

Dasasperi et. al. concluded that CCD imagers give a wider linear range than x-ray film for ECL WBs [18]. Even so, X-ray film detection was used because of analysis speed. When a CCD camera is used, western blots must be treated with ECL reagent and exposed to the camera one at a time. This is time consuming for multiple WBs. When film is used, western blots may be treated with ECL reagent in timed sets and run through the film processor in pairs which is much faster. Additionally, white light densitometer validation is straightforward.

### Strict pTyr WB quantification is impractical

For quantitative bioassays, typically a well characterized substance X is used to create a standard curve to determine the linear range of the assay. The amount of X, or X-like substances in test samples is determined by interpolation from the standard curve. An example is the BCA test for total protein where bovine serum albumin is the standard [19].

For the ECL method of detection, light emission varies with time after application of the luminol reagent as well as with exposure time. For strict quantification, a standard curve would be required for every WB. Algeria et. al. generated standard curves of recombinant proteins IĸBα and p53 using ECL WB. They determined the linear range for IĸBα was 0.097–3.12 ng and for p53 was 1.87–60 ng and were able to quantify changes of these proteins in A431 cultured cells by adding a standard curve to each 1D gel. Changes were confirmed using an independent ELISA test [8].

Running standard curves on every 1D gel for pTyr WB is impractical, and impossible for 2D gels where one sample is loaded per gel. Adding a recombinant pTyr-protein standard to each sample prior to analysis is a practical way to verify the WB method is working properly, to normalize exposure times, and to semi-quantify pTyr proteins in the sample.

### Concerns to be addressed

One concern going forward is whether using a pTyr-RTK fragment as an internal standard for a complex tumor cell lysate would have unintended consequences due to unknown chemical interactions. In a preliminary test, the pA standard showed increased horizontal streaking when added to a human tumor cell lysate before 2D pTyr WB. We hypothesized that tumor binding partners with Src homology 2 domains were interacting with pA during isoelectric focusing. If that hypothesis is correct, it might be necessary to dilute tumor samples out before 2D WB to reduce such pronounced streaking. Additional validation of the pA standard with varied tumor test samples is needed before use.

The Coefficient of Variation (CV = SD/mean *100) for both the 1D and 2D analyses was relatively high, 29–33% for 1D, and 28–30% for 2D runs. The variation between different lanes within a gel for the 1D analyses (S1 Table) was greater than between film exposures suggesting that one source of error is uneven protein transfer across the gel. Further optimization of transblotting is needed to reduce that error.

### Semiquantitative measurements of pTyr-RTK protein in tissues would be useful

Quantification of pTyr-protein levels in tissue samples is complicated because of protein and cellular complexity. Each RTK has a different pTyr activation array; multiple tyrosines may or may not be phosphorylated. For example, EGFR has five Tyr on the C-terminal tail and a sixth site in the kinase domain that become phosphorylated [20]. ALK has three phosphorylation sites in the activation loop and eight elsewhere [21]. The pTyr-protein signal within a tumor lysate will depend on the number of pTyr residues/protein, on pTyr-protein expression within each tumor clone, and on clone abundance within the tumor. Negative control mechanisms, protein Tyr phosphatases for example, affect the pTyr-protein signal as well [22].

Semiquantitative results using pX/pA ratios would indicate if a pTyr-protein were abundantly present in the tissue. Ratios of 10–50 versus 0–2, for example, would likely be indicative of an active clone that might respond to an appropriate inhibitor. Although the pA standard signal is deliberately loaded to be in the linear range of detection, pTyr-RTKs in tissue samples may be in the saturated part of the curve. If so, then the unknown pTyr-protein would be present in higher abundance than the ratio indicates.

### Mass spectrometry provides additional information about ALK phosphorylation

Lemmon and Schlessinger in their review of RTKs note: "The first and primary substrates that RTKs phosphorylate are the receptors themselves" [23]. Results shown in Fig 2 confirm this statement for our *in vitro* reaction.

A review by Sattu et al. summarizes results from four MS labs for which tyrosine residues on the ALK kinase domain are phosphorylated in cultured cells. Within the ALK48 fragment these are: Y1078, Y1092, Y1096, Y1131, Y1278, Y1282 and Y1283 [21]. Identification of only three sites in our experiments suggests that if there are other pTyr sites, their abundance is low, or the phosphate groups are either labile or do not ionize well. Lability of phosphotyrosines during ionization and collision-induced dissociation has been reported and a relatively poor ionization in positive ionization mode has been reported for these phosphopeptides, as compared with their unmodified counterparts [24].

It is not an easy task to identify all phosphotyrosine peptides within one particular experimental setting; many of the challenges for such a task are nicely reviewed by Johnson and White [25]. Rush et. al. showed that Y1078, Y1092, Y1096, Y1131, Y1278 and Y1282 were phosphorylated in ALK [26]. From our experiments, we can confirm that Y1092, Y1096 and Y1282 are phosphorylated, but not Y1283. The MS/MS spectrum that corresponds to peptide ASpYYRK with Y1282 phosphorylated could also match to peptide ASYpYRK, with Y1283 phosphorylated (and neutral loss of the phosphate group). However, to complete a y series, we assigned this MS/MS spectrum to the peptide with ASpYYRK with Y1282 phosphorylated. We always found a peptide TSTIMTDYNPNpYC(#)FAGK TSTIM(ox)TDYNPNpYC(#)FAGK, with Y1096 phosphorylated, but Y1092 was always unphosphorylated. However, after we enriched the phosphopeptides using TiO2 (Thermo Fisher Scientific Inc.), Ni-NTA (Thermo Fisher Scientific Inc.), and Glygen enrichment tips, we also found peptide TSTImTDpYNPNYC(#)FAGK with Y1092 phosphorylated and Y1096 unphosphorylated. Therefore, Y1092 is phosphorylated, but at a very low percentage, as compared with Y1096.

It has been suggested that within ALK’s YxxxYY motif, the first Y is almost exclusively phosphorylated [27] and that this motif, although identical to the one from insulin receptor substrate, has a different phosphorylation pattern [27]. Within ALK, YxxxYY motif corresponds to Y1278, Y1282 and Y1283. Our data contradicts this statement. While we do not find solid evidence that Y1278 is phosphorylated, we do find that Y1282 is clearly phosphorylated, and Y1283 is not. While these findings contradict Donella-Deana and colleagues’ work [27] who stated that only Y1278 is phosphorylated, they do agree with Rush and colleagues’ work [26], who found that both Y1278 and Y1282 (but not Y1283) are phosphorylated.

Contradictory results were also observed in regard to Y1131. Rush and colleagues found Y1131 phosphorylated (peptide GLGHGAFGEVpYEGQVSGMPNDPSPLQVAVK) [26]. In our experiments, we found exactly the same peptide, but not phosphorylated. While in our (unenriched and enriched) experiments Y1131, Y1092 and Y1278 were found not phosphorylated, we also looked for these phosphopeptides in the raw data. Many times, manual search allows one to extract additional information. Still, we did not find any additional evidence for additional phosphorylated peptides. Since the experiments in Donella were done in vitro, but used a non-MS approach, while the experiments in Rush were performed by MS analysis where all phosphopeptides were investigated, we believe that the studies in Rush are closer to reality. It is possible that since we performed our experiments in vitro (only on the truncated ALK), the phosphorylation is preferential to Y1096 and Y1282 and is perhaps less abundant on Y1092, Y1131 and Y1278.

In our MS analysis, we found solid evidence that Y1096 (multiple pTyr unenriched and enriched experiments) and Y1282 (1 experiment) are phosphorylated, and, after enrichment, found that Y1092 was phosphorylated. The 2D pTyr WB spot however, was streaky (see Fig 7) instead of resolving into distinct charge isoforms, suggesting charge microheterogeneity. We hypothesize that the chemiluminescent response of our phosphotyrosine marker is mainly linked to phosphorylation at Y1096 with lesser antibody binding to Y1282, Y1092, and possibly to other tyrosine residues phosphorylated at a lower level.

### SDS compatibility is essential for analysis of membrane proteins

The Andersons first described 2D compatibility with SDS for the carrier ampholine method of IEF in tube gels, which was later confirmed and proven quantitative by our lab [11, 13]. They showed that as IEF proceeds overnight, SDS is stripped off proteins to make micelles with NP-40, a non-ionic detergent. The micelles migrate to the extreme acid end of the tube gel where they form a bulb that may be discarded. Classic 2D SDS PAGE is technically more laborious than the commonly used immobilized pH gradient (IPG) strip method [28]. Unfortunately, IPG strips are incompatible with SDS and do not resolve large membrane proteins. In 2009, Rabilloud summarized the importance of using SDS to solubilize membrane proteins for any kind of analysis. He showed that heavily glycosylated membrane proteins migrate as fuzzy 2D spots, as observed here for pTyr-EGFR, and concluded that SDS-only separations are the safe way to analyze membrane proteins [29].

These results show that standardized 2D SDS PAGE, where SDS is used in both IEF and MW dimensions, in combination with ECL WB can resolve important membrane proteins that are intractable to other methods. Adding a pTyr-protein internal standard such as pA to every sample allows for the comparison of pTyr proteins between samples using 1D/2D pTyr WB. Combining results from this and other specialized protein/mRNA analysis methods including MS, immunohistology, and RNA-seq may be the way forward to find biomarkers for Precision Medicine.

## Materials and methods

### A431 cell lysate containing pTyr-EGFR

Two different lots of pTyr-EGFR positive control sample (pE) were purchased from Exalpha (Shirley, MA, Cat # X1003, A431 cell lysates stimulated by EGF, lots 10852 and 13639), referred to as lots 1 and 2. The lots were used without dilution.

### Recombinant human active protein kinase ALK48 fragment

ALK wt (HIS-tag, product No 1048-0000-1, MW 47,991 daltons) internal fragment with amino acids L1066-S1437 (Gen Bank entry NM_004304.3, S3 Fig) was purchased from ProQinase, GmbH, Freiburg, Germany.

### Antibodies

Primary antibodies used included mouse monoclonal anti-pTyrosine, clone PY20 (ExAlpha, Cat # X1021, Lot # 12175) and monoclonal rabbit EGF Receptor (D38B1) XP^®^ antibody (Cell Signaling, Danvers, MA, Cat # 4267S, Lot # 8). The strong pTyr signal in the post-kinase reaction vs the pre-kinase reaction along with the 1D linear WB response validated the anti-pTyr antibody for this study. MS confirmed that the post-kinase ALK48 contained pTyr. Secondary antibodies used included sheep anti-mouse IgG-HRP (GE Healthcare, Cat# NA931, RRID:AB_772210, Lot # 9729340) and sheep anti-rabbit IgG-HRP (GE Healthcare, Cat# NA934, RRID:AB_772206, Lot # 9495175).

### Tumor sample preparation

Patient tumor samples were purchased from a tissue bank, Bio-IVT. All steps were performed on ice. The samples (~1g each) were cut in half. One half was frozen immediately on dry ice for later use. The other half rinsed with 2 ml of ice-cold tris buffered saline (TBS, containing 150 mM NaCl, 50 mM Tris-Cl, pH 7.5), then placed in a motorized glass homogenizer. Two ml of Osmotic Lysis Buffer (10 mM Tris, pH 7.4, and 0.3% SDS) was added that contained protease inhibitors, phosphatase inhibitors, and OmniCleave endonuclease. 100X Protease Inhibitor Stock Solution contained 20 mM AEBSF (# 101500), 1 mg/ml leupeptin (# L2884), 0.36 mg/ml E-64 (# E3132), 500 mM EDTA (# 34103), and 5.6 mg/ml benzamidine (# B6506) all purchased from MilliporeSigma. Two Phosphatase Inhibitors cocktails from EMD Millipore were used, one for protein serine/threonine phosphatases (#524624) and one for protein tyrosine phosphatases (#524625). OmniCleave™ Endonuclease from Lucigen (#OC7850K) is a purified enzyme from a recombinant E. coli strain that works in the presence of SDS. It was used to reduce sample viscosity by degrading native DNA and RNA to di-, tri- and tetra-nucleotides. Three ml of SDS boiling buffer (5% SDS, 10% glycerol and 60 mM Tris, pH 6.8) was added and the tissue homogenized on ice with up and down motions until much of the tissue was dissolved and the remainder dispersed. The tube was placed in a boiling water bath for 5 min until the solution clarified. A protein determination was performed using the BCA method, and the samples diluted to 10 mg/ml before adding 5% beta-mercaptoethanol (BME) and dividing into 100 ul aliquots stored at -80°C. Note that BME interferes with the BCA assay.

### Preparation of pA

#### Tyrosine phosphorylation of ALK48 starting material

Twenty μl aliquots of ALK48 (ProQinase lot 005, 673 ng/μl) containing 13.5 μg of protein were auto-phosphorylated by reaction with 2 mM ATP at 30°C for one hour in kinase activation buffer specified by ProQinase on two different days to give lots 1 and 2. The activation buffer contained 50 mM HEPES, pH 7.5, 100 mM NaCl, 2 mM DTT, 7.5 mM MgCl_2_ and 7.5 mM MnCl. The tyrosine-phosphorylated form of ALK48 is called pTyr-ALK48.

#### Sulfhydryl blocking of pTyr-ALK48

Cysteine sulfhydryl groups on pTyr-ALK48 were alkylated by iodoacetamide (IAA) from Thermo Scientific according to the manufacturer’s instructions. The sample was pretreated with TCEP-HCP for 1 hour at 55°C and reacted with IAA for 30 min at 55°C protected from light. The reaction was stopped by addition of SDS Buffer containing 5% BME. The final carbamydomethyl-cysteine standard is designated with an SB suffix (sulfhydryl blocked) i.e. pTyr-ALK48-SB (pA). The reaction was performed on 5 μg of pTyr-ALK48 Lot 1 and 13.4 μg of Lot 2 on separate days.

### 1D SDS PAGE

Proteins were electrophoretically separated using 10% SDS PAGE gels poured in-house with 30% acrylamide stock and 0.8% N,N'-Methylene Bisacrylamide crosslinker, both from National Diagnostics (Atlanta, GA). A stacker gel was added with 15-well comb to provide wells for loading samples. SDS slab gel electrophoresis was carried out for 4 hours at 15 mA/gel. The following proteins (Sigma Chemical Co., St. Louis, MO and EMD Millipore, Billerica, MA) were run in one lane as molecular weight standards: myosin (220,000), phosphorylase A (94,000), catalase (60,000), actin (43,000), carbonic anhydrase (29,000), and lysozyme (14,000).

### 2D SDS PAGE

Two dimensional SDS PAGE (2D SDS PAGE) was performed according to the method of O'Farrell [30] essentially as described by Lopez-Coral et. al. [31]. Isoelectric focusing was performed in 4% acrylamide tube gels of 2.3 mm internal diameter, polymerized with 1.5% piperazine diacrylamide (Sigma-Aldrich, St. Louis, MO) added to 30% acrylamide stock plus 9M urea (MP Biomedicals Solon, OH); 10% IGEPAL (Sigma-Aldrich); and 2% ampholines (Serva Electrophoresis Heidelberg, Germany). Samples were dissolved in 1:1 SDS buffer: Urea Buffer before being loaded at the top (basic end) of the tube gels. Voltage was applied for 20,000 volt-hrs (1000v for 20 hr).

Tube gels were extruded by air pressure applied with a syringe into a screw-top test tube containing SDS buffer. After equilibration for 10 minutes in equilibration buffer containing 10% glycerol, 50 mM dithiothreitol, 2.3% SDS and 0.0625 M tris, pH 6.8, each tube gel was sealed to the top of a stacking gel overlaying a 10% acrylamide slab gel (1.0 mm thick). SDS slab gel electrophoresis was carried out for about 5 hours at 25 mA/gel. Molecular weight markers described for 1D SDS PAGE were run in a lane on the basic edge of the slab gels. Stained gels were dried between sheets of cellophane paper with the acid edge to the left. One μg of an IEF internal standard, tropomyosin, was added to every sample for quality control. Tropomyosin migrates as a doublet with lower polypeptide spot of MW 33,000 and pI 5.2.

### Western Blotting (WB)

After slab gel electrophoresis, the gels were placed in transfer buffer (10 mM CAPS, pH 11.0, 10% methanol) and transblotted onto PVDF membranes (Immobilon P, 0.45 μm, MilliporeSigma, St. Louis, MO) overnight at 200 mA, 4°C and approximately 100 volts/ two gels in a model EBU-100 apparatus from CBS Scientific (Del Mar, CA). PVDF membranes were stained with Coomassie blue (Sigma-Aldrich) in a 50% methanol, 0.12% Coomassie blue dye (adjusted for dye) for 5 minutes on an orbital shaker, then destained in a solution of 50% methanol by shaking for 1.5 minutes. Finally, the blots were rinsed twice for 1 minute with ultrapure water and placed on a filter paper sheet to air dry. Images of dried Coomassie-stained PVDF patterns were recorded by scanning with a desktop scanner. For storage, the stained blots were covered with filter paper sheets and placed between poster board supports. Coomassie staining does not interfere with subsequent WB.

Coomassie blue stained PVDF membranes were wet in 100% methanol to remove the stain, rinsed briefly in tween-20 tris buffer saline (TTBS), and blocked for two hours in 5% nonfat dry milk (NFDM) diluted in TTBS. The blots were incubated overnight on an orbital shaker at room temperature with pTyr or EGFR primary antibody diluted 1:1000 in 2% NFDM TTBS. One blot was incubated with EGFR primary antibody diluted 1:10,000 in 2% NFDM TTBS. The blots were rinsed 3 x 10 minutes in TTBS and shaken with secondary antibody anti-mouse IgG HRP (for pTyr blots) or anti-rabbit IgG HRP from sheep (for EGFR blots) diluted 1:2000 for 2 hours. Finally, the blots were treated with Pierce ECL reagent (ThermoFisher) and exposed to Kodak BioMax MR film (8" x 10") from ThermoFisher for 3 and 10 minutes followed by film development with an automatic Medical Film Processor SRX-101A (Konica Minolta, Tokyo, Japan).

### Densitometry and image quantification

Films were scanned on a laser densitometer (Model PDSI, Molecular Dynamics Inc, Sunnyvale, CA) calibrated to be linear over 0–3.0 OD units. Phoretix 1D software (TotalLab, UK) was used for SDS PAGE while their 2D software, Progenesis SameSpots/PG240, was used for quantification of 2D spots. The background density algorithm was “average on boundary”.

### Mass spectrometry to determine tyrosine phosphorylation sites

For mass spectrometry analysis, the gel spots were digested with trypsin and the peptides mixture was then extracted and cleaned as described by Channaveerappa et. al. The nanoliquid chromatography-tandem mass spectrometry (nanoLC-MS/MS) experiments were performed using a NanoAcquity UPLC coupled with a QTOF Xevo G2 [14, 32]. The MS raw data were processed and converted into pkl files using the ProteinLynxGlobal Server version 2.4 (Waters Corporation, Milford, MA) and the database search was performed using Daemon software, version 2.5.1. (Matrix Science, London, UK). Search parameters used were as follows: propionamide for cysteine, carbamidomethyl cysteine, methionine oxidation and phosphotyrosine were chosen as variable modifications. The precursor ion and the product ion mass tolerance were set to 0.5 Da and 0.8 Da respectively. The pkl files were then searched against NCBI nr human database, with one tryptic missed cleavage. Customized database search against the pALK was also performed. Additional searches with less stringent parameters (i.e. up to 3 missed cleavages or 1 and 2 13C) were also performed. Identification of the pY was performed both at the peptide level, and also at the amino acid level. When more than one Tyr residue was on a peptide, information from the b, a and y ions, as well as from the neutral loss of the phosphate group were also used to confirm the location of the phosphorylated amino acid. Verification of the phosphopeptides in the raw MS/MS spectra for the location of the phosphate group was also performed and the pY peptides were confirmed.

## Supporting information

S1 Fig Preliminary 2D SDS PAGE testing of c-Jun N-terminal kinase 1 (JNK1) to determine suitability as a pTyr protein standard. The two rightmost spots on the Coomassie stained PVDF membrane (A) do not react on the western blot (B). The two leftmost spots on the western blot were not on the Coomassie stained membrane and are thus unexplained.
(TIF)

S2 Fig Preliminary 1D SDS PAGE testing for chemically tyrosinylated bovine serum albumin (pTyr BSA) to determine suitability as a pTyr-protein standard. The extreme heterogeneity of the pTyr signal renders this protein useless as a standard.
(TIF)

S3 Fig Amino acid sequence of recombinant protein ALK48. The amino acid sequence of ALK48 is in blue (residues 1065 to 1428) relative to that of UniProtKB—Q9UM73 (ALK_HUMAN) in black provided by ProQinase. The recombinant product also contained inert FLAG tag at the beginning and polyHis tag at the end to facilitate purification (not shown).
(TIF)

S4 Fig The MS spectra of the precursor ions (inbox) with m/z of 999.05 (2+) and 1039.04 (2+) that correspond to unphosphorylated peptide TSTIMTDYNPNYC(#)FAGK and its phosphorylated counterpart TSTIMTDYNPNpYC(#)FAGK. Note that C(#) represents cysteine modified by acrylamide (propionamide) and pY corresponds to phosphorylated tyrosine residue. Fragmentation of the precursor ions in MSMS produced a set of product ions that correspond to the unphosphorylated (top) and phosphorylated (bottom) peptide. The tyrosine residue that is phosphorylated is Y1096, while Y1092 is not.
(TIF)

S5 Fig MS spectrum of the precursor ion (inbox) with m/z of 434.26 (2+) that corresponds to phosphorylated peptide ASpYYRK. Note that pY corresponds to phosphorylated tyrosine residue. Fragmentation of the precursor ion in MSMS produced a set of product ions that correspond to the phosphorylated (bottom) peptide. The tyrosine residue that is phosphorylated is Y1282, while Y1283 is not.
(TIF)

S6 Fig MS spectrum of the precursor ion (inbox) with m/z of 1038.61 (2+) that corresponds to phosphorylated peptide TSTIMTDpYNPNYC(#)FAGK Note that C(#) represents cysteine modified by acrylamide (propionamide) and pY corresponds to phosphorylated tyrosine residue. Fragmentation of the precursor ion in MSMS produced a set of product ions that correspond to the phosphorylated peptide. The tyrosine residue that is phosphorylated is Y1092, while Y1096 is not.
(TIF)

S1 Table Band density values and pE/pA density ratios for Run 1 (pE Lot 1) and Run 2 (pE Lot 2) shown in Fig 5. The pE/pA ratios were calculated using the 1 ng pA band density (n = 2 lanes) on the same gel. In Run 1, this value was 1000 for the 3 min 1787 for the 10 min. For Run 2, the value was 1152 for the 3 min and 2110 for the 10 min. Average values contain ±SD; CV, coefficient of variation = SD/mean *100.
(DOCX)

S1 Dataset Raw data from 1D and 2D western blots underlying all findings. (XLSX)

S1 Raw images Original images behind all figures and data analysis. (PDF)
