# Supplementary figures and images for: Preparation of a phosphotyrosine-protein standard for use in semiquantitative western blotting with enhanced chemiluminescence
Source: PLoS One. 2020 Jun 18;15(6):e0234645. doi: 10.1371/journal.pone.0234645 (PMC7302481; doi:10.1371/journal.pone.0234645)

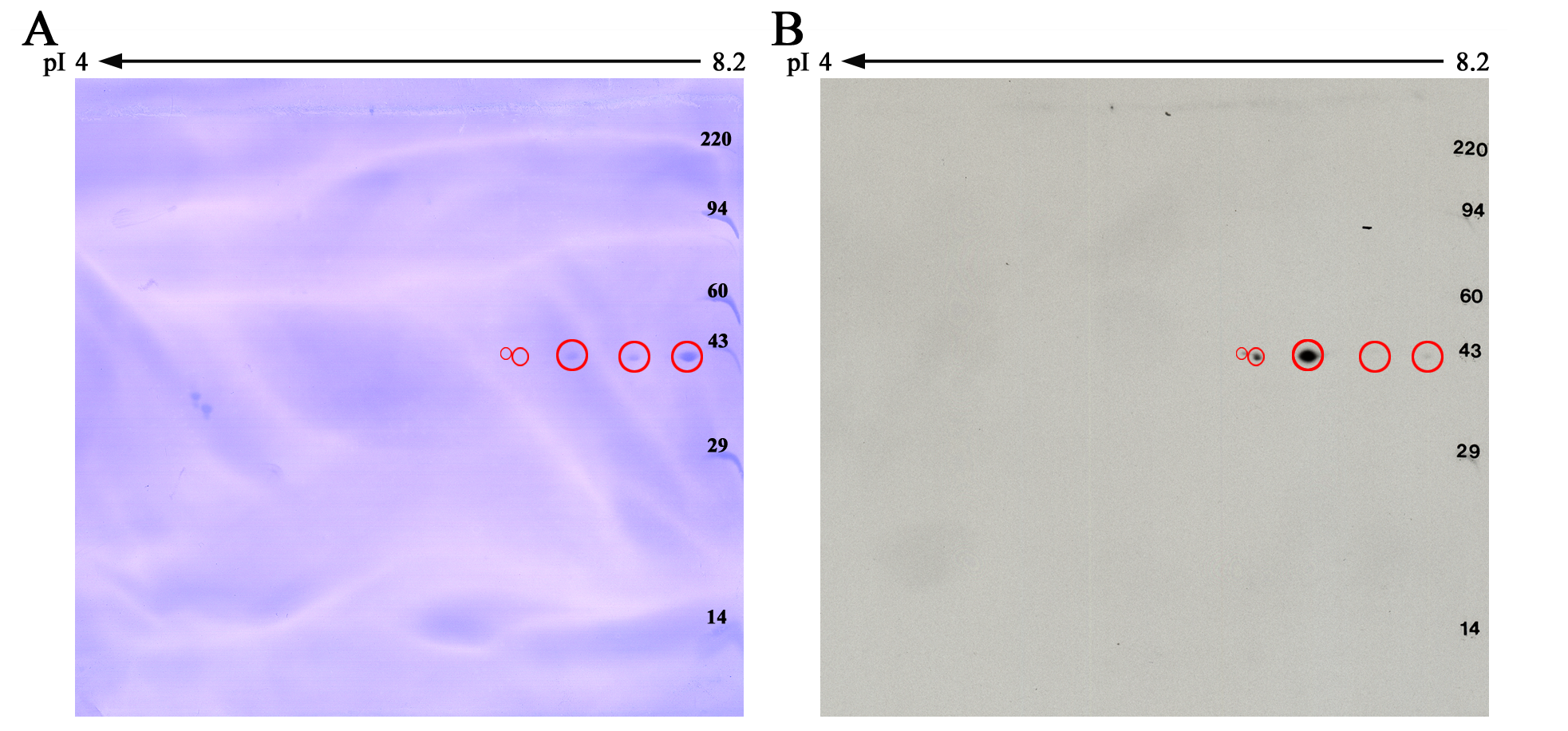

Supplement: S1 Fig — The two rightmost spots on the Coomassie stained PVDF membrane (A) do not react on the western blot (B). The two leftmost spots on the western blot were not on the Coomassie stained membrane and are thus unexplained. (TIF) [file pone.0234645.s001.tif]

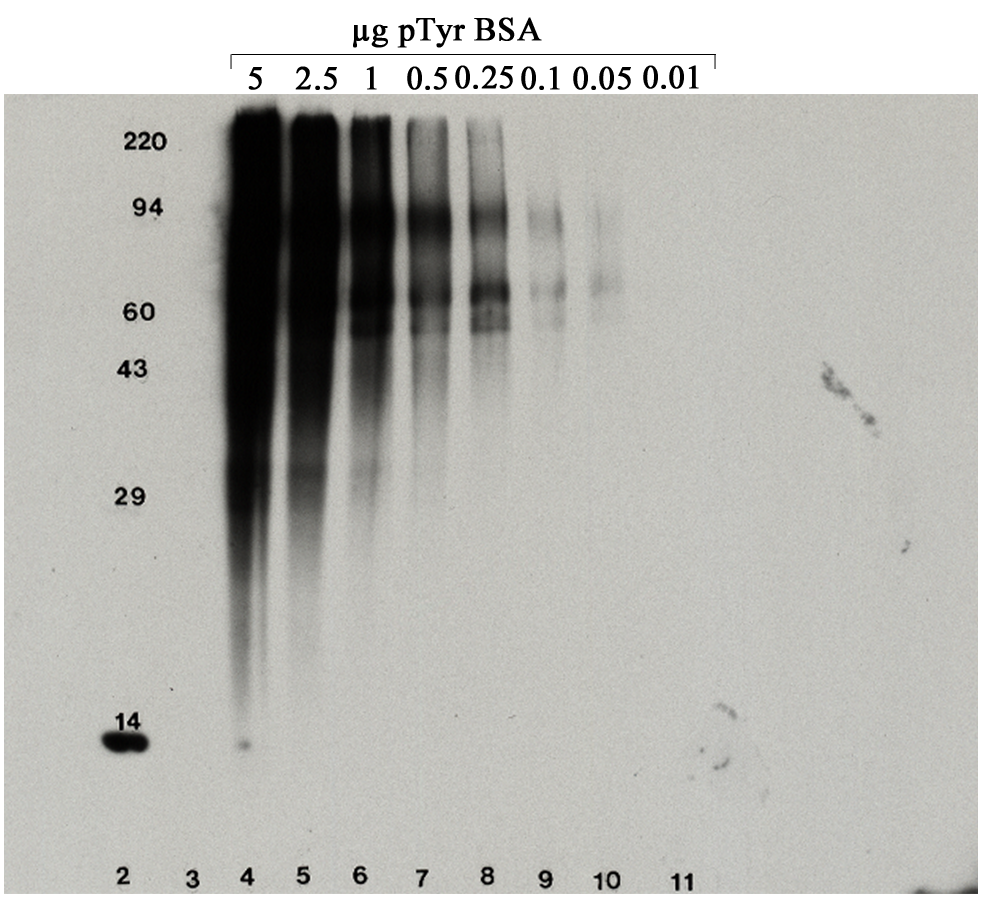

Supplement: S2 Fig — The extreme heterogeneity of the pTyr signal renders this protein useless as a standard. (TIF) [file pone.0234645.s002.tif]

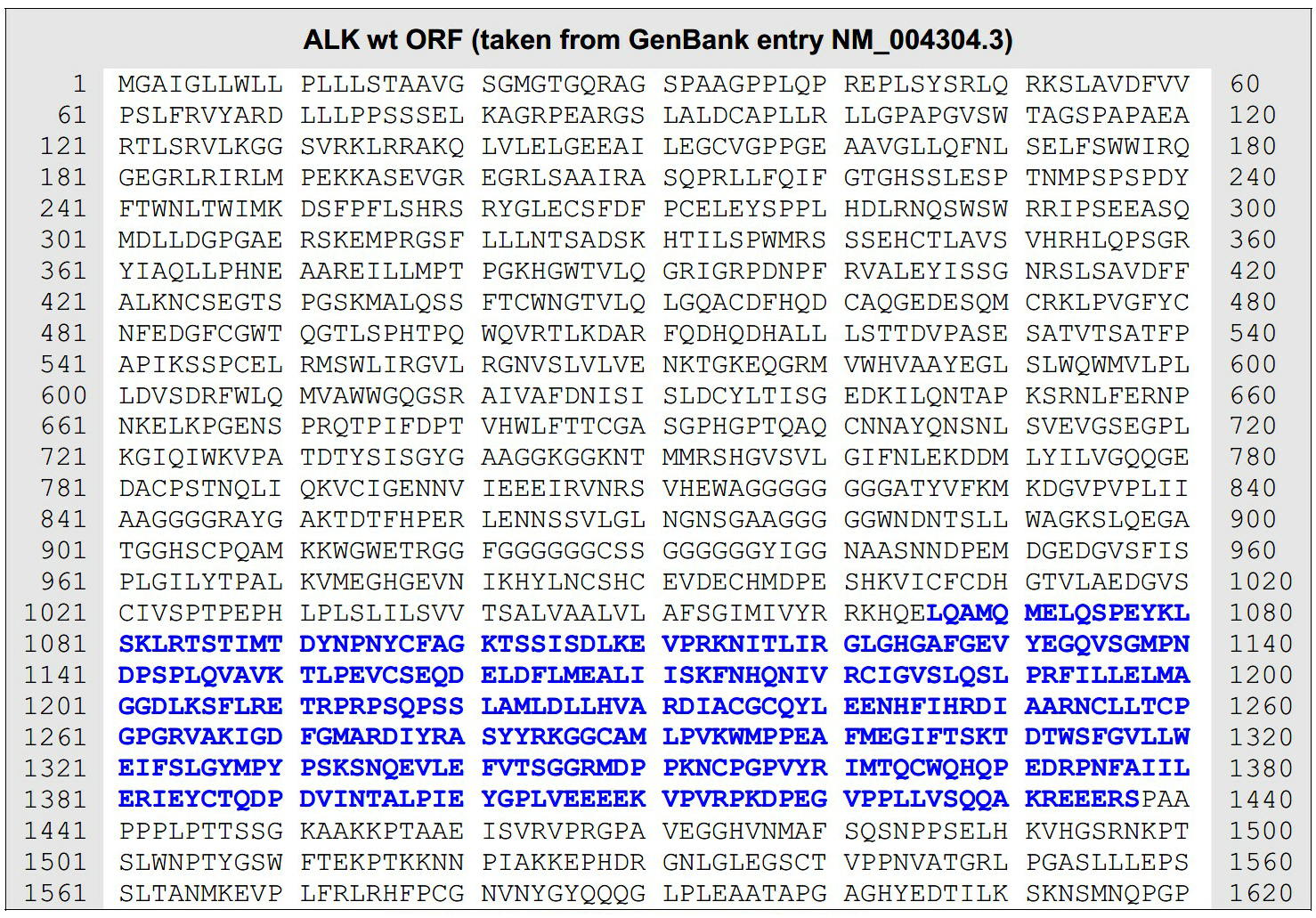

Supplement: S3 Fig — The amino acid sequence of ALK48 is in blue (residues 1065 to 1428) relative to that of UniProtKB—Q9UM73 (ALK_HUMAN) in black provided by ProQinase. The recombinant product also contained inert FLAG tag at the beginning and polyHis tag at the end to facilitate purification (not shown). (TIF) [file pone.0234645.s003.tif]

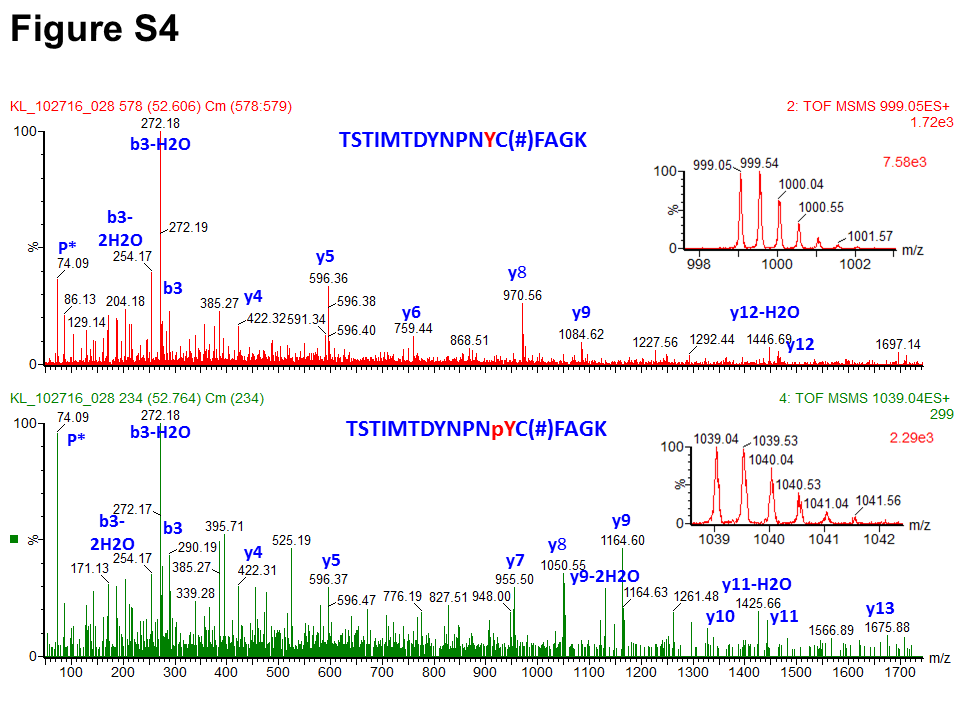

Supplement: S4 Fig — Note that C(#) represents cysteine modified by acrylamide (propionamide) and pY corresponds to phosphorylated tyrosine residue. Fragmentation of the precursor ions in MSMS produced a set of product ions that correspond to the unphosphorylated (top) and phosphorylated (bottom) peptide. The tyrosine residue that is phosphorylated is Y1096, while Y1092 is not. (TIF) [file pone.0234645.s004.TIF]

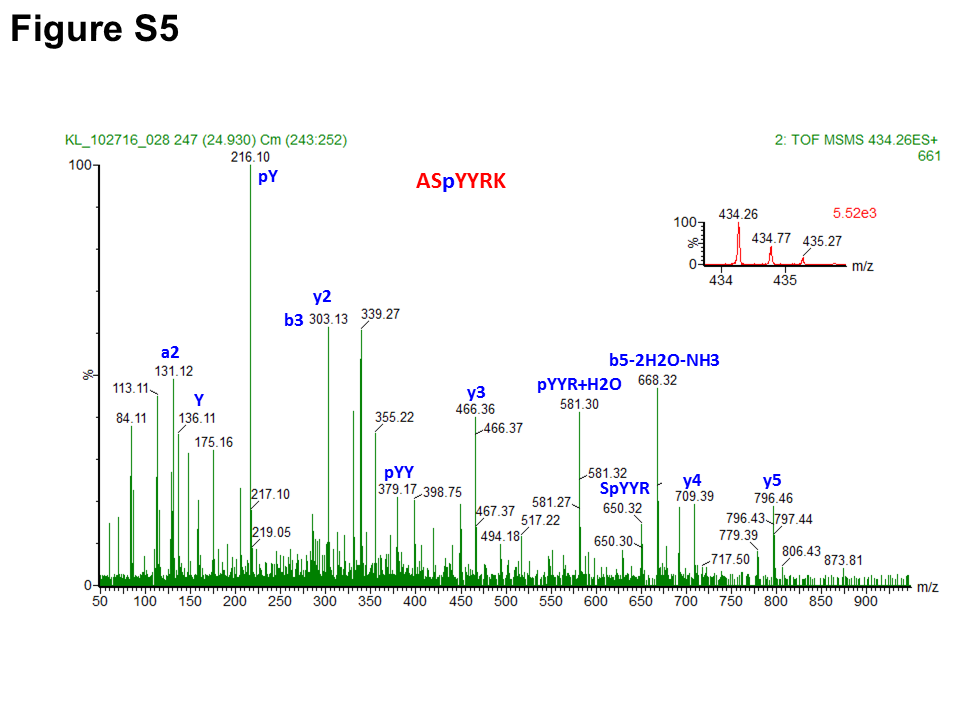

Supplement: S5 Fig — Note that pY corresponds to phosphorylated tyrosine residue. Fragmentation of the precursor ion in MSMS produced a set of product ions that correspond to the phosphorylated (bottom) peptide. The tyrosine residue that is phosphorylated is Y1282, while Y1283 is not. (TIF) [file pone.0234645.s005.TIF]

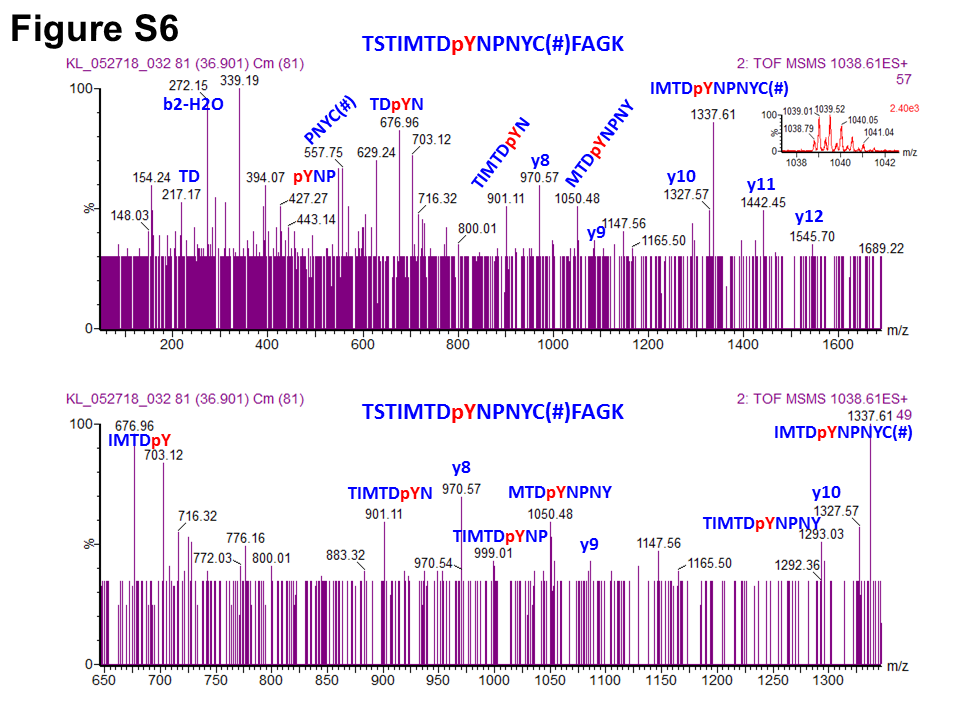

Supplement: S6 Fig — Fragmentation of the precursor ion in MSMS produced a set of product ions that correspond to the phosphorylated peptide. The tyrosine residue that is phosphorylated is Y1092, while Y1096 is not. (TIF) [file pone.0234645.s006.TIF]
