# Supplementary material for: Preparation of a phosphotyrosine-protein standard for use in semiquantitative western blotting with enhanced chemiluminescence
Source: PLoS One. 2020 Jun 18;15(6):e0234645. doi: 10.1371/journal.pone.0234645 (PMC7302481; doi:10.1371/journal.pone.0234645)
Supplement: S1 Table — The pE/pA ratios were calculated using the 1 ng pA band density (n = 2 lanes) on the same gel. In Run 1, this value was 1000 for the 3 min 1787 for the 10 min. For Run 2, the value was 1152 for the 3 min and 2110 for the 10 min. Average values contain ±SD; CV, coefficient of variation = SD/mean *100. (DOCX) [file pone.0234645.s007.docx]

**S1 Table. Band density values and pE/pA density ratios for Run 1 (pE Lot 1) and Run 2 (pE Lot 2) shown in Fig 5.**

The pE/pA ratios were calculated using the 1 ng pA band density (n=2 lanes) on the same gel. Average values contain ±SD; CV, coefficient of variation = SD/mean *100.
