# Supplementary material for: Preparation of a phosphotyrosine-protein standard for use in semiquantitative western blotting with enhanced chemiluminescence
Source: PLoS One. 2020 Jun 18;15(6):e0234645. doi: 10.1371/journal.pone.0234645 (PMC7302481; doi:10.1371/journal.pone.0234645)
Supplement: S1 Raw images — (PDF) [file pone.0234645.s009.pdf]

L3 Lung tumor, pTyr WB

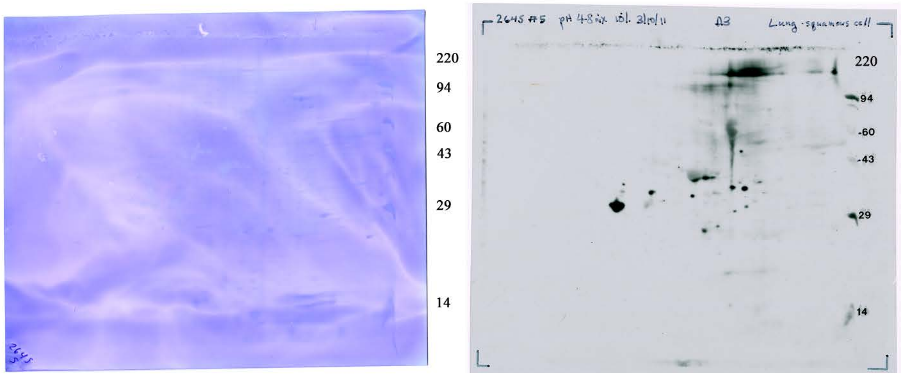

L3 Lung NAT, pTyr WB

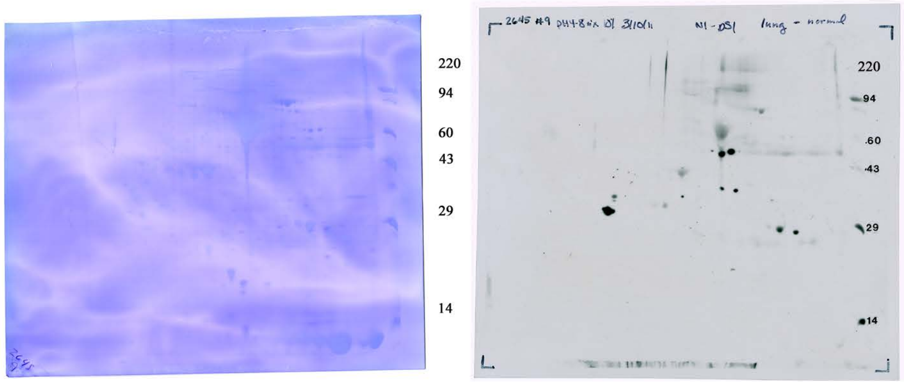

L3 Lung tumor, EGFR WB

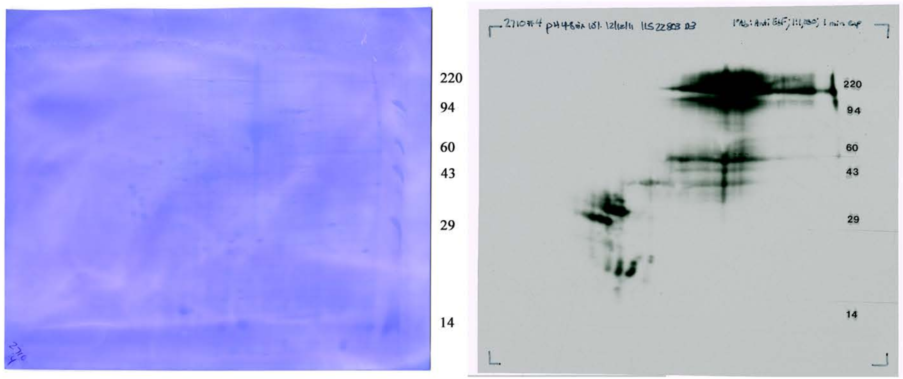

L3 Lung NAT, EGFR WB

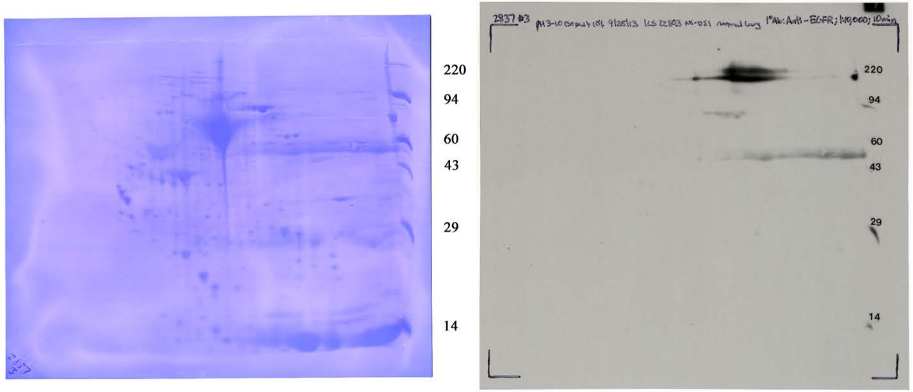

PVDFs and anti-pTyr or anti-EGFR western blots loaded with lung tumor tissue or normal adjacent tissue samples. The blots are used in Fig 1 in the main text. PVDFs were stained with Coomassie blue and films were developed with an automatic Medical Film Processor SRX-101A.

ALK

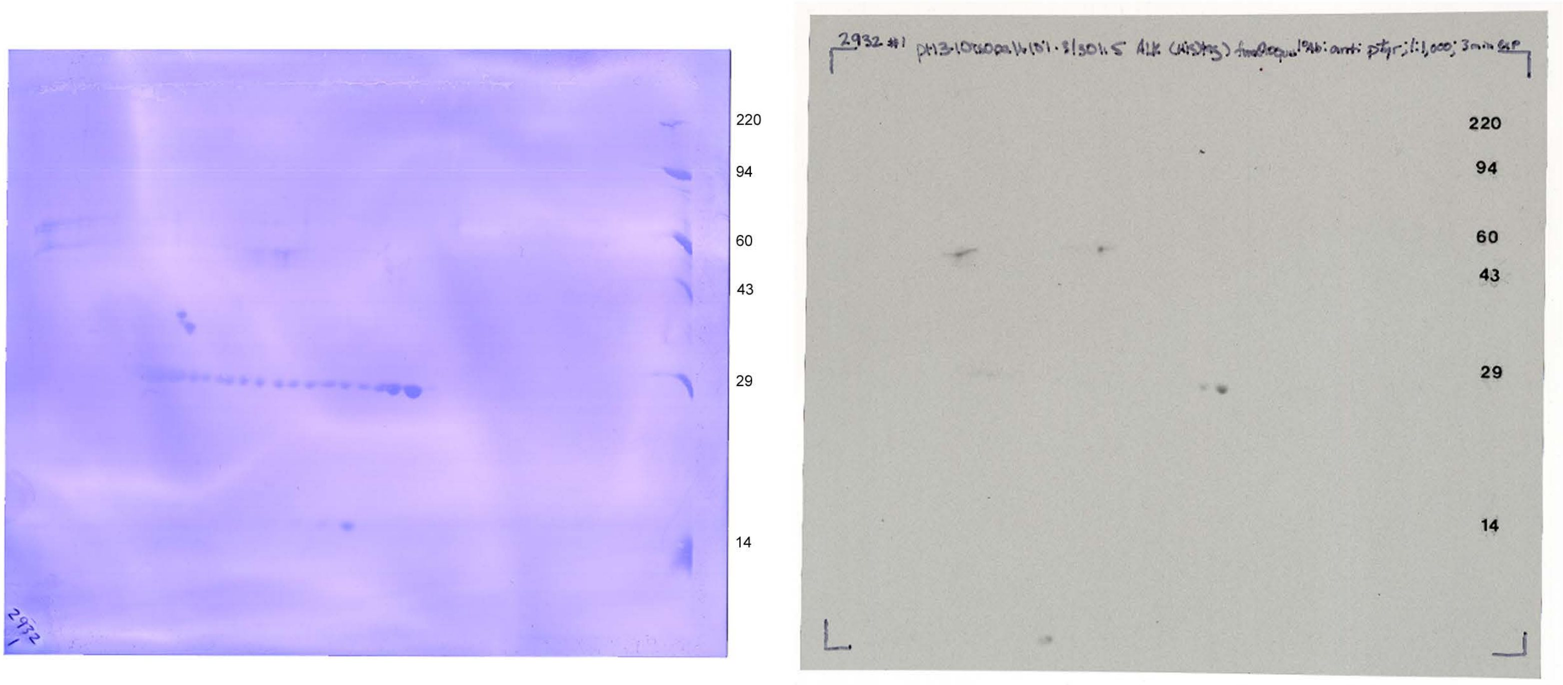

pALK

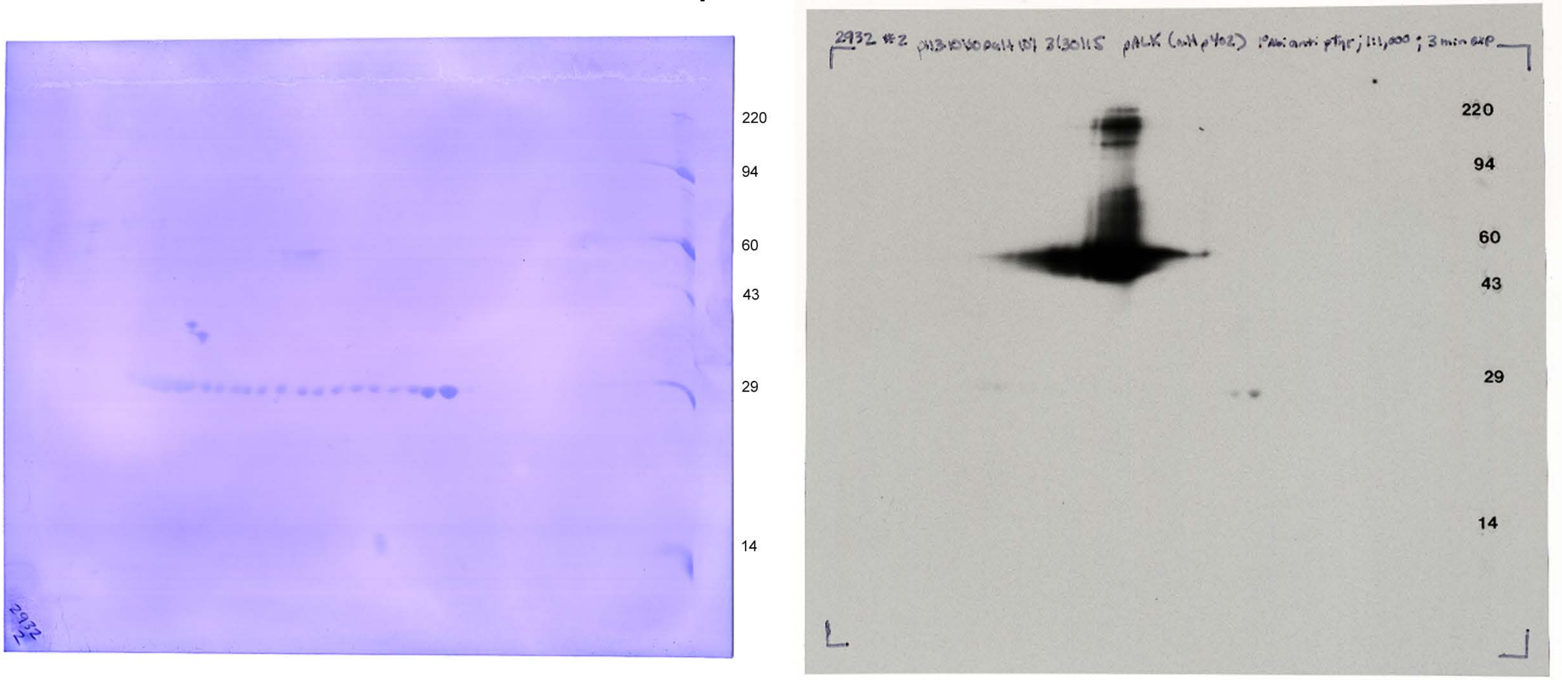

PVDFs and anti-pTyr western blots from Fig 2 in the main text. PVDFs were stained with Coomassie blue and films were developed with an automatic Medical Film Processor SRX-101A.

pALK

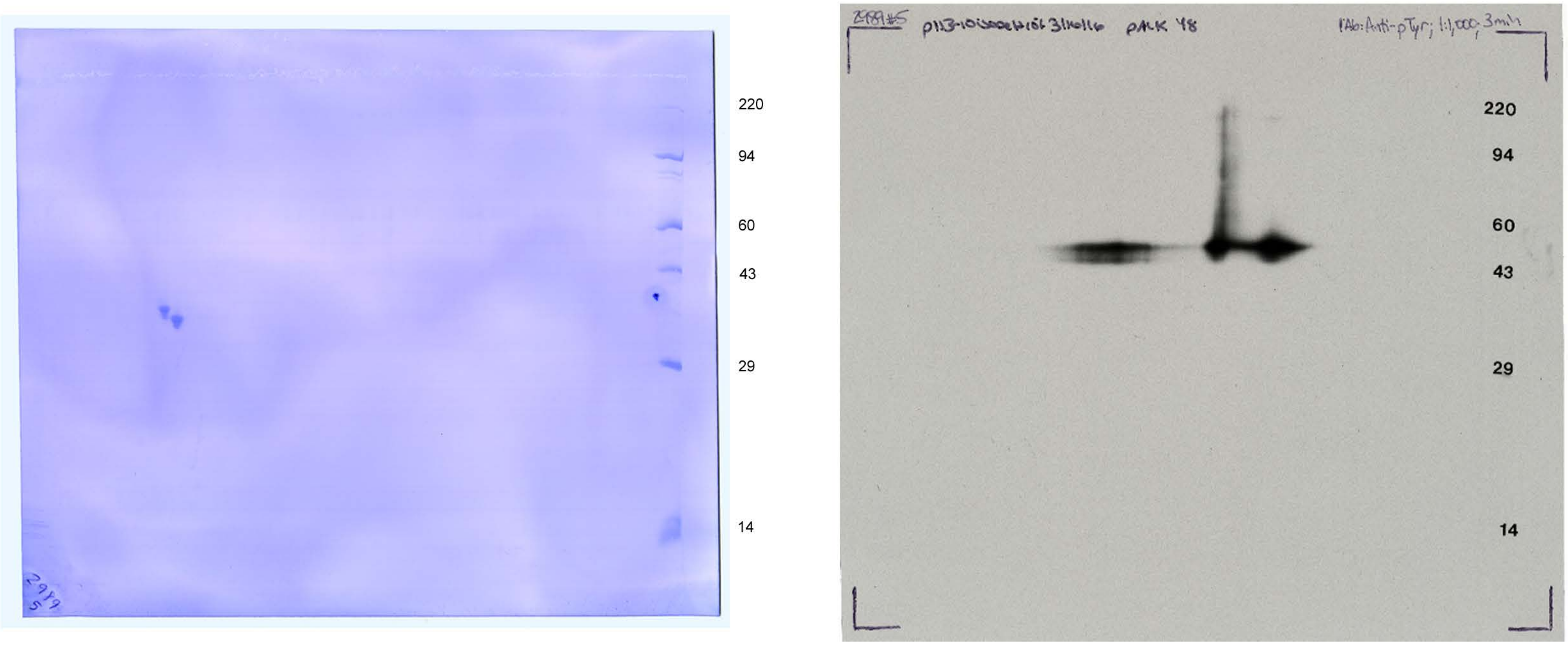

pALK SB

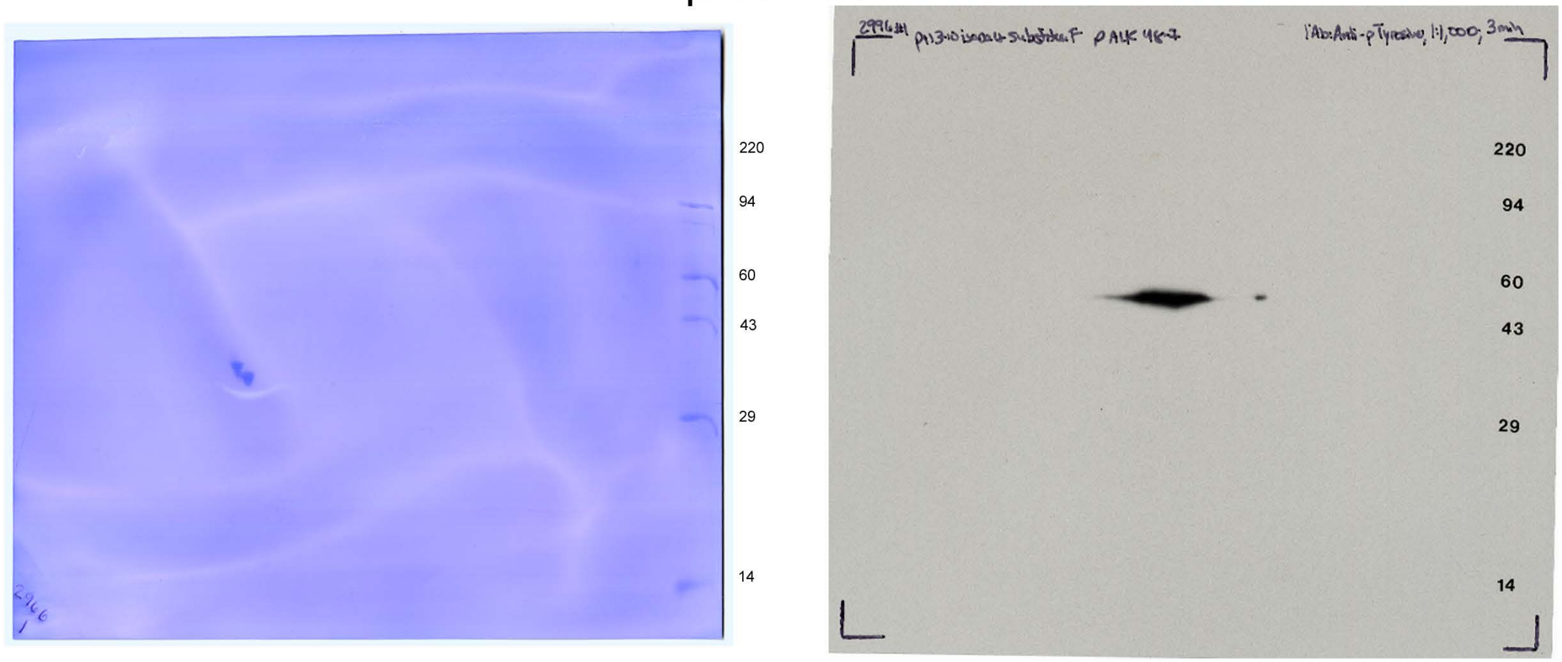

PVDFs and anti-pTyr western blots from Fig 3 in the main text. PVDFs were stained with Coomassie blue and films were developed with an automatic Medical Film Processor SRX-101A.

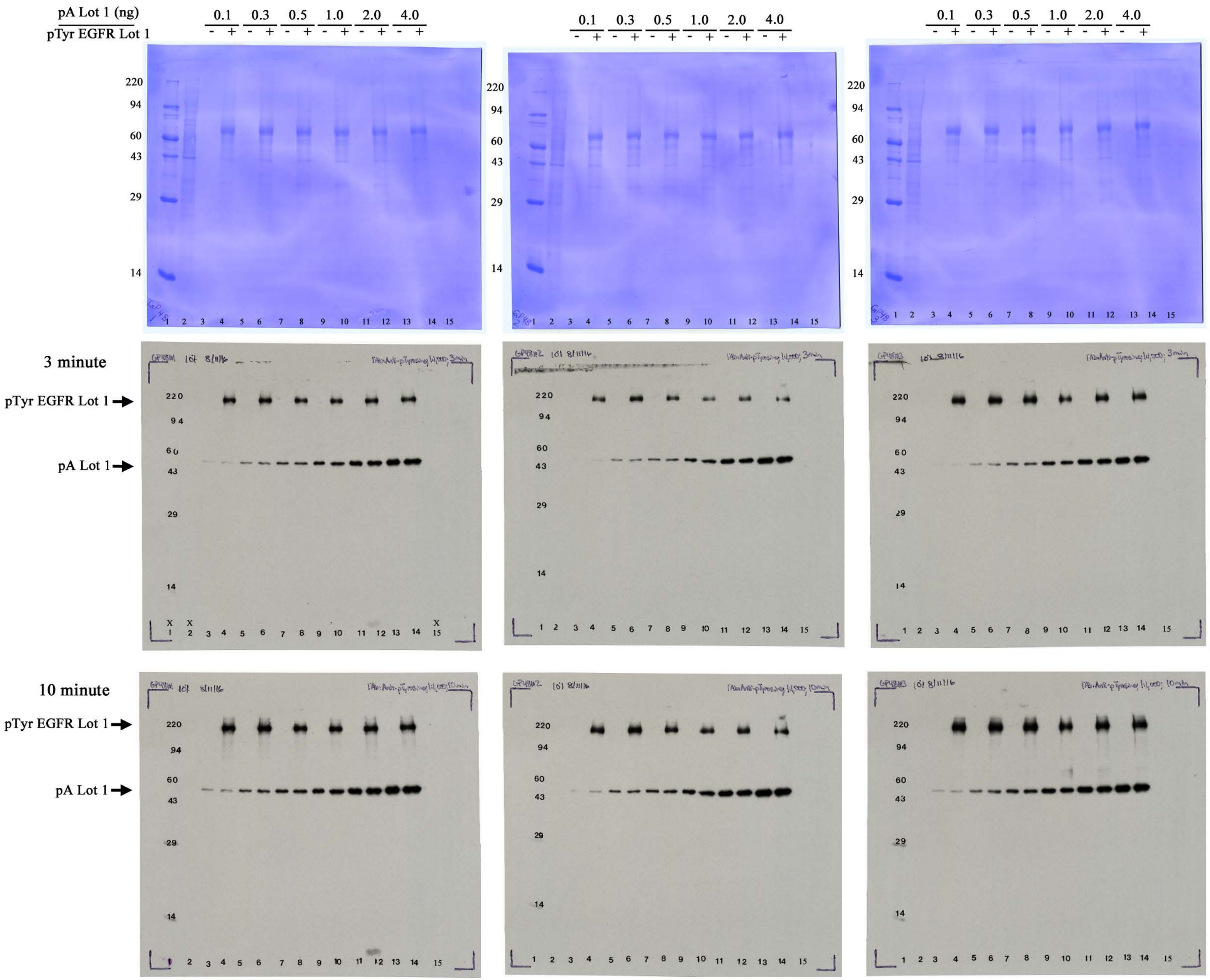

PVDFs and anti-pTyr western blots from triplicate gels loaded with pTyr EGFR Lot 1 and pA Lot 1. The 3 minute anti-pTyr western blot from the first run (far left) is used in Figs 5-6 in the main text. All blots were used in the data analysis done for Table 1. PVDFs were stained with Coomassie blue and films were developed with an automatic Medical Film Processor SRX-101A.

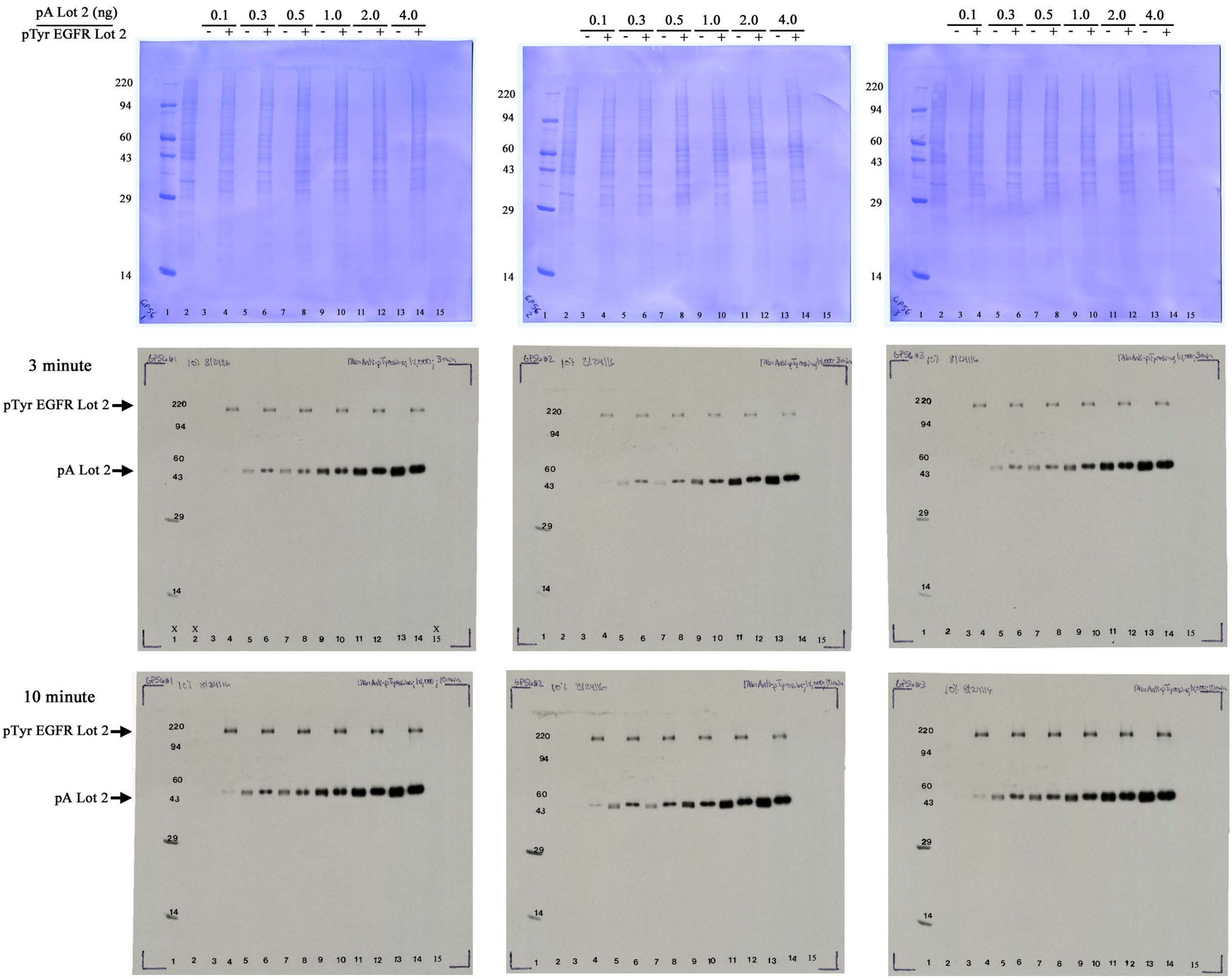

PVDFs and anti-pTyr western blots from triplicate gels loaded with pTyr EGFR Lot 2 and pA Lot 2. The 3 minute anti-pTyr western blot from the first run (far left) is used in Figs 5-6 in the main text. All blots were used in the data analysis done for Table 1. PVDFs were stained with Coomassie blue and films were developed with an automatic Medical Film Processor SRX-101A.

pE/pA Lot 1

3 minute

10 minute

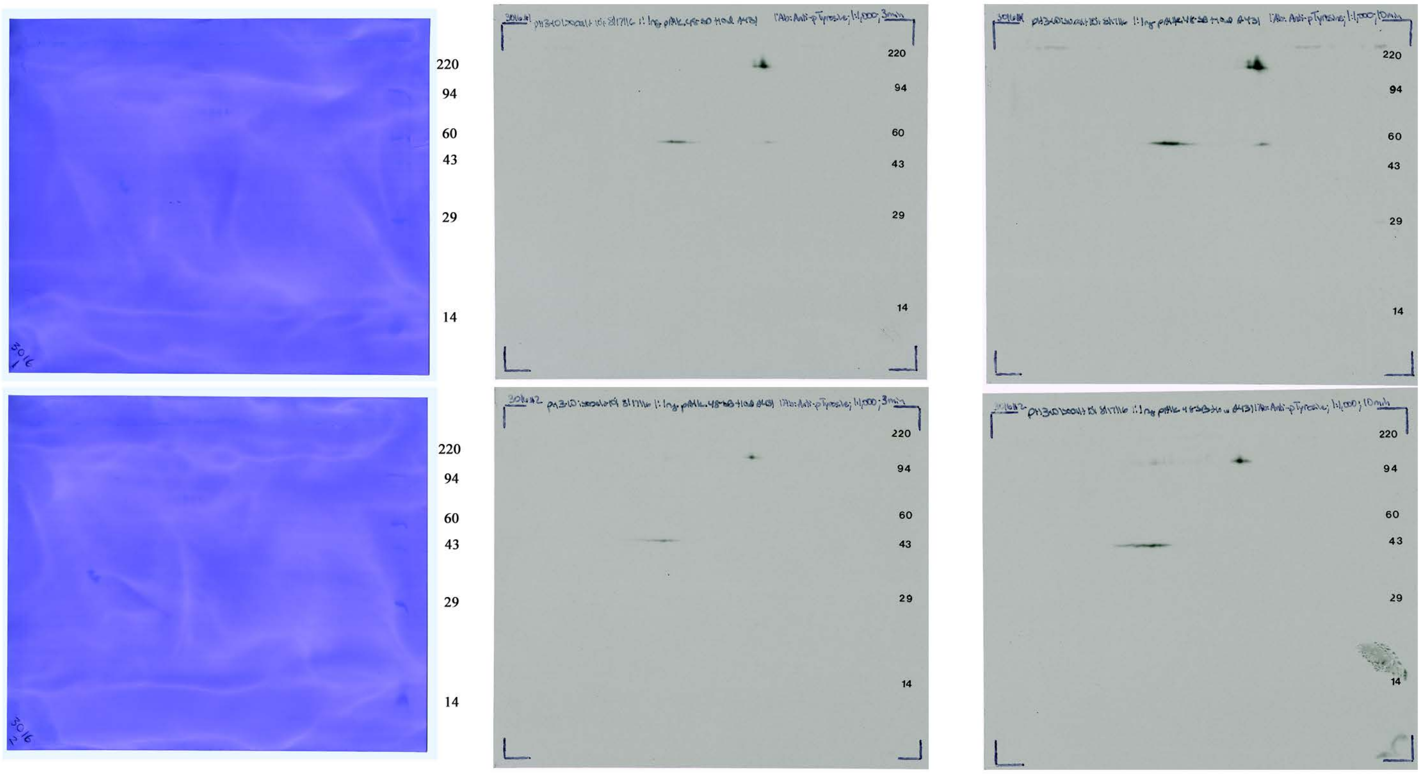

pE/pA Lot 2

3 minute

10 minute

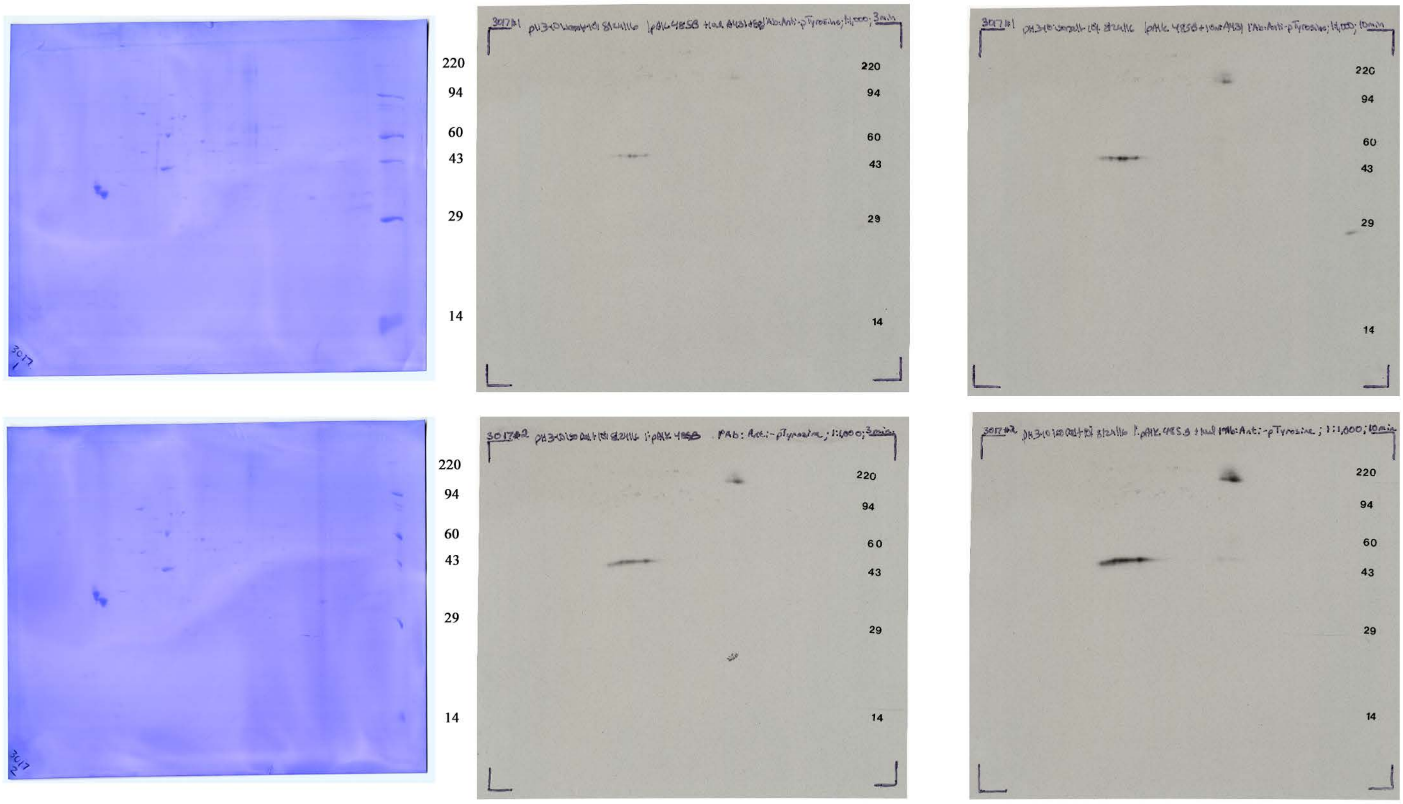

PVDFs and anti-pTyr western blots from duplicate gels loaded with pE/pA Lot 1 or 2 according to the loading conditions in Table 2. All blots were used in the data analysis for Table 3 and Fig 8. PVDFs were stained with Coomassie blue and films were developed with an automatic Medical Film Processor SRX-101A.

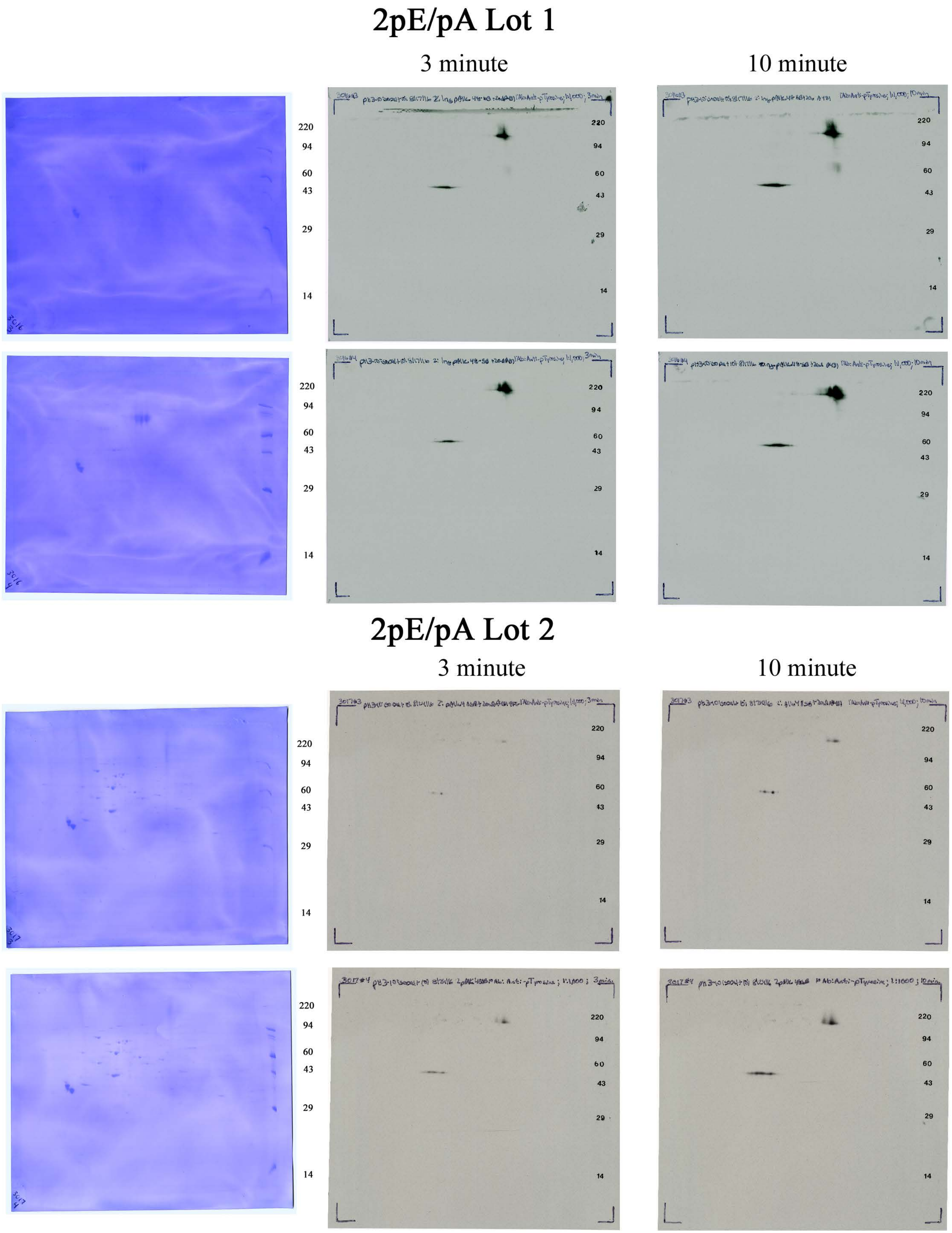

PVDFs and anti-pTyr western blots from duplicate gels loaded with 2pE/pA Lot 1 or 2 according to the loading conditions in Table 2. All blots were used in the data analysis for Table 3 and Fig 8. PVDFs were stained with Coomassie blue and films were developed with an automatic Medical Film Processor SRX-101A.

pE/2pA Lot 1

3 minute | 10 minute

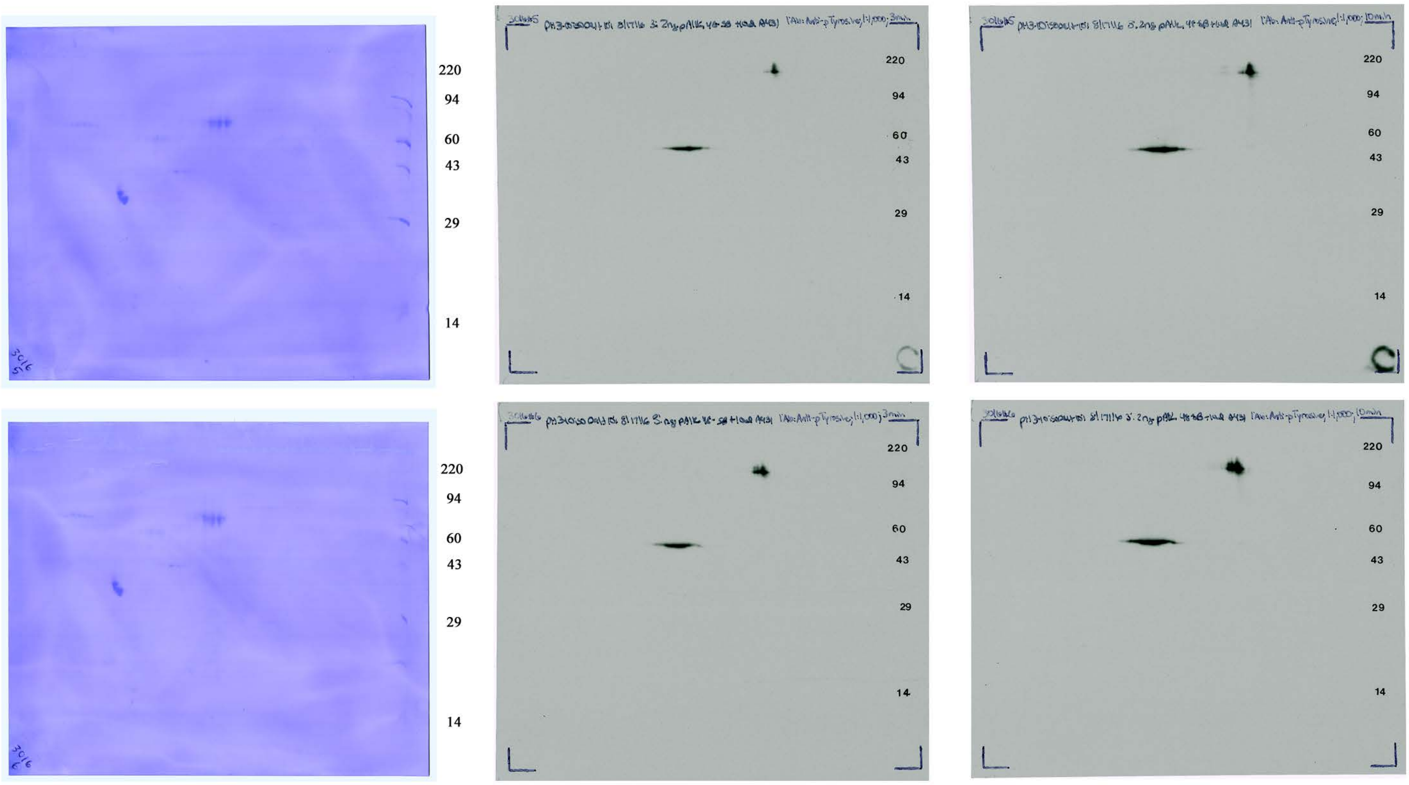

pE/2pA Lot 2

3 minute | **10 minute**

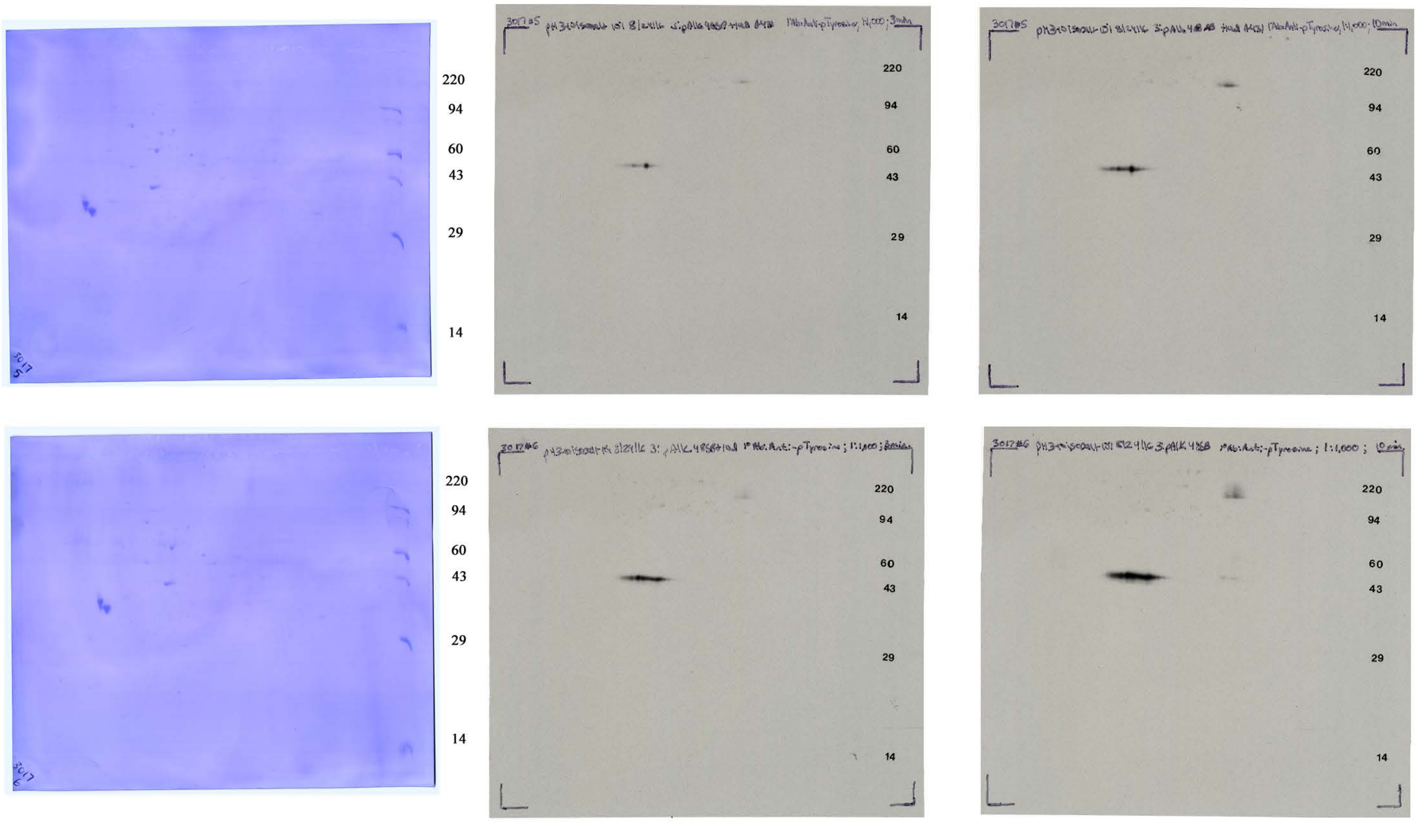

PVDFs and anti-pTyr western blots from duplicate gels loaded with pE/2pA Lot 1 or 2 according to the loading conditions in Table 2. All blots were used in the data analysis for Table 3 and Fig 8. PVDFs were stained with Coomassie blue and films were developed with an automatic Medical Film Processor SRX-101A.

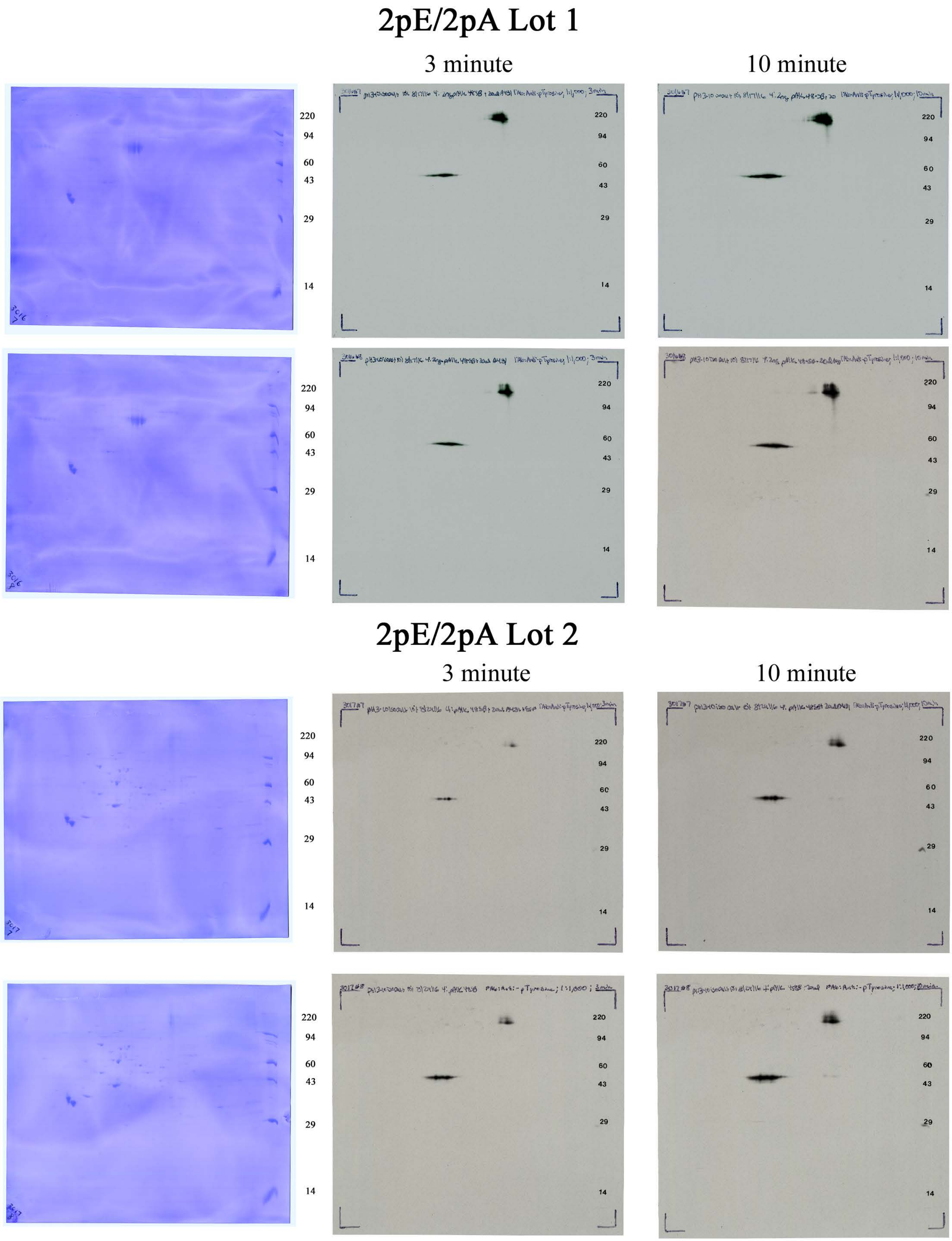

PVDFs and anti-pTyr western blots from duplicate gels loaded with 2pE/2pA Lot 1 or 2 according to the loading conditions in Table 2. The 10 minute western blots from the second gel of each lot were used in Fig 7. All blots were used in the data analysis for Table 3 and Fig 8.PVDFs were stained with Coomassie blue and films were developed with an automatic Medical Film Processor SRX-101A.

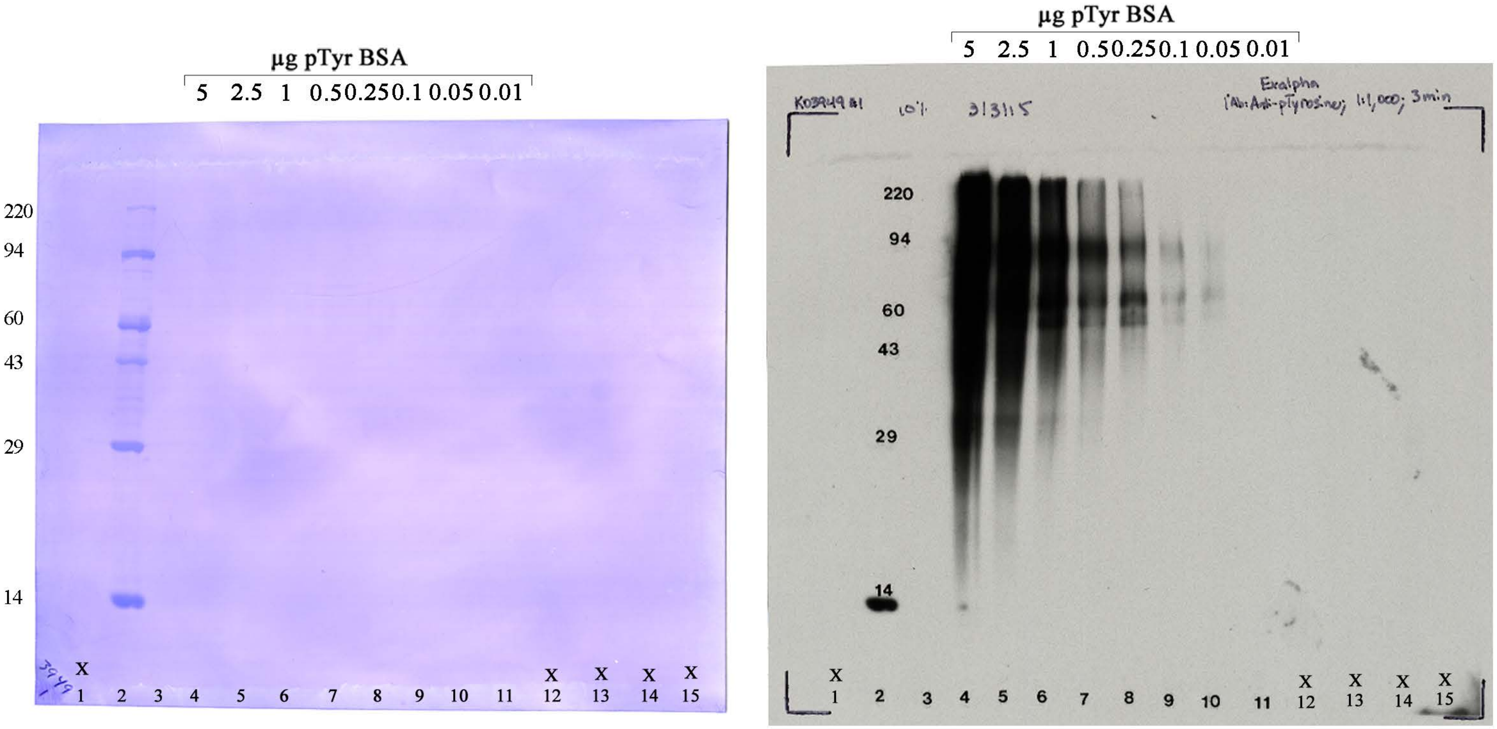

PVDF and anti-pTyr western blot from S1 Fig. The PVDF was stained with Coomassie blue and the film was developed with an automatic Medical Film Processor SRX-101A.
